# Unsupervised discovery of dynamic cell phenotypic states from transmitted light movies

**DOI:** 10.1371/journal.pcbi.1009626

**Published:** 2021-12-30

**Authors:** Phuc Nguyen, Sylvia Chien, Jin Dai, Raymond J. Monnat, Pamela S. Becker, Hao Yuan Kueh

**Affiliations:** 1 Department of Bioengineering, University of Washington, Seattle, Washington, United States of America; 2 Molecular Engineering and Sciences Institute, University of Washington, Seattle, Washington, United States of America; 3 Division of Hematology, University of Washington, Seattle, Washington, United States of America; 4 Department of Laboratory Medicine and Pathology, University of Washington, Seattle, Washington, United States of America; 5 Department of Genome Sciences, University of Washington, Seattle, Washington, United States of America; 6 Institute for Stem Cell and Regenerative Medicine, University of Washington, Seattle, Washington, United States of America; 7 Clinical Research Division, Fred Hutchinson Cancer Research Center, Seattle, Washington, United States of America; 8 Division of Hematology/Oncology, Department of Medicine, University of California, Irvine, California, United States of America; 9 Chao Family Comprehensive Cancer Center Cancer Research Institute, University of California, Irvine, California, United States of America; University at Buffalo - The State University of New York, UNITED STATES

## Abstract

Identification of cell phenotypic states within heterogeneous populations, along with elucidation of their switching dynamics, is a central challenge in modern biology. Conventional single-cell analysis methods typically provide only indirect, static phenotypic readouts. Transmitted light images, on the other hand, provide direct morphological readouts and can be acquired over time to provide a rich data source for dynamic cell phenotypic state identification. Here, we describe an end-to-end deep learning platform, UPSIDE (Unsupervised Phenotypic State IDEntification), for discovering cell states and their dynamics from transmitted light movies. UPSIDE uses the variational auto-encoder architecture to learn latent cell representations, which are then clustered for state identification, decoded for feature interpretation, and linked across movie frames for transition rate inference. Using UPSIDE, we identified distinct blood cell types in a heterogeneous dataset. We then analyzed movies of patient-derived acute myeloid leukemia cells, from which we identified stem-cell associated morphological states as well as the transition rates to and from these states. UPSIDE opens up the use of transmitted light movies for systematic exploration of cell state heterogeneity and dynamics in biology and medicine.

## Author summary

The human body contains hundreds of different cell types, each with distinctive identities and functions. Identifying these cellular identities and functional states is one of the great challenges in contemporary biology. We have addressed this challenge by developing UPSIDE, a machine learning approach for discovering cell types and cell states from time-resolved live cell imaging data, which provides a richly detailed source of information. Key distinguishing features of UPSIDE include the ability to directly learn cell type-defining features from brightfield images without prior knowledge; and an ability to visualize and interpret these machine-learned features to understand why they were chosen. UPSIDE can distinguish different human blood cell types despite their visual similarity and can use morphologic clues to identify and follow cell states. In a disease-relevant example, we used UPSIDE to identify stem-like and more mature cell subpopulations in patient-derived acute myelogenous leukemia cells, and to determine the speed at which these cell types inter-convert. UPSDIDE provides a general-purpose tool for the unbiased identification and analysis of cell types and state transitions in heterogeneous cell populations.

This is a *PLOS Computational Biology* Methods paper.

## Introduction

Cells maintain and switch between distinct phenotypic states in a dynamic manner. Identifying these states and understanding the basis for and dynamics by which they interconvert is a central challenge in biology. Modern single-cell analysis methods, such as single cell RNA sequencing and multiparameter flow cytometry or mass cytometry [1–5], are widely used to define cell states in heterogeneous populations; while powerful, these methods provide incomplete readouts of cell phenotypes, and typically do not report on stability or transition dynamics. Transmitted light microscopy images directly reveal cell morphology and have historically formed the basis for identifying cell types and cell states in diverse fields, ranging from cell biology to neuroscience [6,7]. These images can then be acquired at successive timelapse intervals and over long times, with minimal phototoxicity and without prior labeling or genetic manipulation. The resultant live cell movies can reveal additional information about the dynamics of these cell phenotypic states.

Cell phenotypes have traditionally been identified by the visual inspection and interpretation of transmitted light or electron micrographic images. The advent of modern machine learning, however, is enabling high-throughput automated analysis of cell morphology, and is opening possibilities for using deep learning for systematic, unbiased extraction of dynamic cell morphological states from these imaging data sets [8,9]. However, current tools are still limited in their ability to perform such analyses. First, current deep learning pipelines for cell image analysis rely heavily on predetermined knowledge to generate classification training datasets, or on large sets of heuristic formulations to capture the diversity of cell shapes and morphologies [10–13]. When examining novel biological processes with minimal to no preconceived information, it can be difficult for investigators to determine what the important labels are without manual intervention and feature selection. Second, current machine learning pipelines generate features that are often not readily interpretable. A variety of unsupervised methods can generate reduced dimensionality representations from complex data, including principal component analysis (PCA), adversarial autoencoders [14], generative adversarial network [15,16], and self-supervised deep learning approaches [9,17]. However, these methods are limited in their ability to generate interpretable morphological features that allow for further investigation and understanding of the machine-identified cell states. Finally, current movie analysis methods cannot infer state transition dynamics from live cell movies in an automated, systematic manner [18]. Cell state transitions are typically observed from trajectories of single cells; however, despite recent advances [19], current tracking algorithms still typically require considerable parameter adjustment and manual error correction for generation of cell trajectories [20].

Here, we present an end-to-end deep learning method for elucidating cell phenotypic states and their dynamics from brightfield movies of living cells. This method, termed UPSIDE (for Unsupervised Phenotypic State IDEntification), is designed to facilitate unsupervised discovery of cellular phenotypic states, elucidation of morphological features that define these states, and inference of state transition dynamics. UPSIDE segments cells directly from brightfield images, then utilizes the variational autoencoder architecture (VAE) [21] to learn intuitive latent features that can be clustered to reveal distinct morphological states, and also decoded to extract human-interpretable meaning. In order to demonstrate use and versatility of UPSIDE, we first analyzed static images of a collection of distinct blood cell types, to identify morphological features that distinguish these different cell types. We then analyzed live imaging movies of leukemic cells from an acute myeloid leukemia (AML) patient to identify morphologically-distinct cell states associated with stemness, and determined the rates of transition to and from these states. These results demonstrate the utility of UPSIDE as a tool for unbiased exploration of cellular states and their dynamics from large, time-resolved imaging datasets.

## Results

### Description of the UPSIDE platform

**Fig 1:**
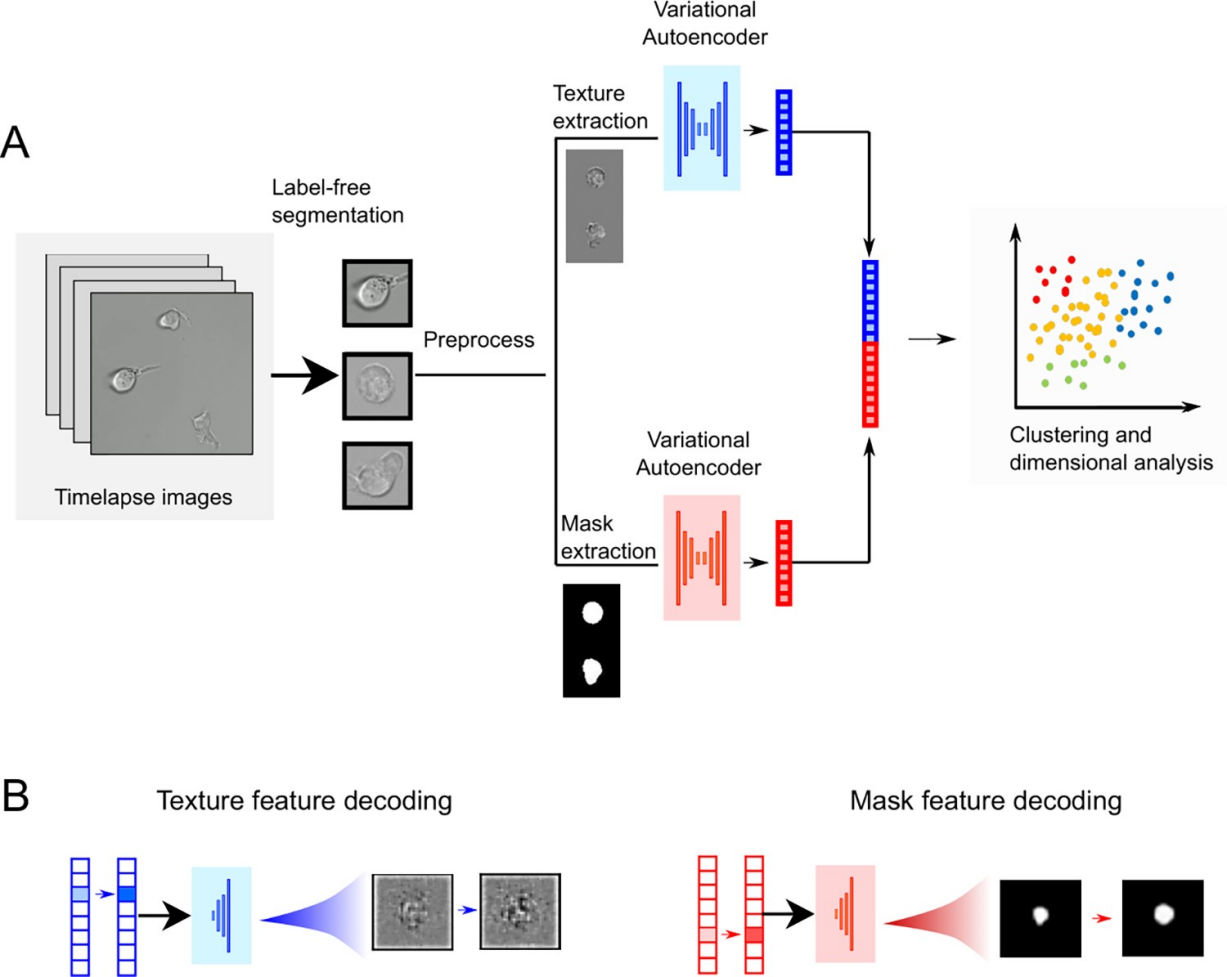
Description of the UPSIDE workflow. (A) Single cells are segmented directly from brightfield images and deep learning UNET architecture to predict synthetic fluorescent images [23]. Segmented cells are then pre-processed to generate separate mask and texture images, which are then used to concurrently train two variational autoencoders (VAEs). The shape and texture encodings learnt by these two VAEs are then concatenated and used for downstream data analysis. (B) Encoded latent vectors are then decoded into a shape and texture image to aid the interpretation of the encoded features.

UPSIDE is designed to be a versatile machine-learning pipeline for unsupervised exploration of cell morphological states in transmitted light images, and subsequent elucidation of their transition dynamics from movies (Fig 1A, see *Methods* section for detailed description of the pipeline). In this pipeline, cells are first segmented using a convolutional neural network that converts brightfield images of unlabeled cells into synthetic fluorescent images of cytoplasm for segmentation [22]. This neural network is trained using a set of images of cells stained for their cytoplasm (S1 Fig). This approach allows the network to autonomously tailor its parameters, and to accommodate a wide range of different cell types in order to optimize performance without human input. Dead cells and other debris are eliminated from identified cell sub-images through a convolutional classifier trained on cells from the same data set that were manually identified as being dead or alive (S2 Fig).

UPSIDE then learns morphological features of identified live cells using a variational autoencoder (VAE) architecture. To enhance the sensitivity of UPSIDE towards learning true cell-state or cell-type defining morphological features, we incorporated two computational procedures into our analysis pipeline. First, we rotated cells to align their major axes vertically, then reflected them to ensure an identical left-right skewing, to ensure cell encodings are invariant to rotation and reflectional transformations. Second, we trained two VAEs in parallel, one that takes a binary cell mask, and another that takes a normalized grayscale image containing cell textural features (see methods). We did so to ensure that both shape and textural morphological features of imaged cells are adequately utilized for feature encoding. The learned mask and textural encodings were then weighed using a coefficient, Ω, then concatenated for subsequent clustering and dimensionality reduction. We note that Ω can be adjusted depending on application to capture the relative importance of shape and textural features to underlying cell heterogeneity.

Latent representations of cells are then clustered using the Louvain method [23] and represented on a 2D plane using the uniform manifold and projection algorithm (UMAP) [24]. Finally, mask and texture vectors are subject to decoding through variation of magnitudes of specific features or groups of features, followed by generation of synthetic images in observable image space (Fig 1B). This approach allows latent features to be visually displayed for human inspection and interpretation.

### UPSIDE uncovers distinguishing morphological features between heterogeneous blood cell types

**Fig 2:**
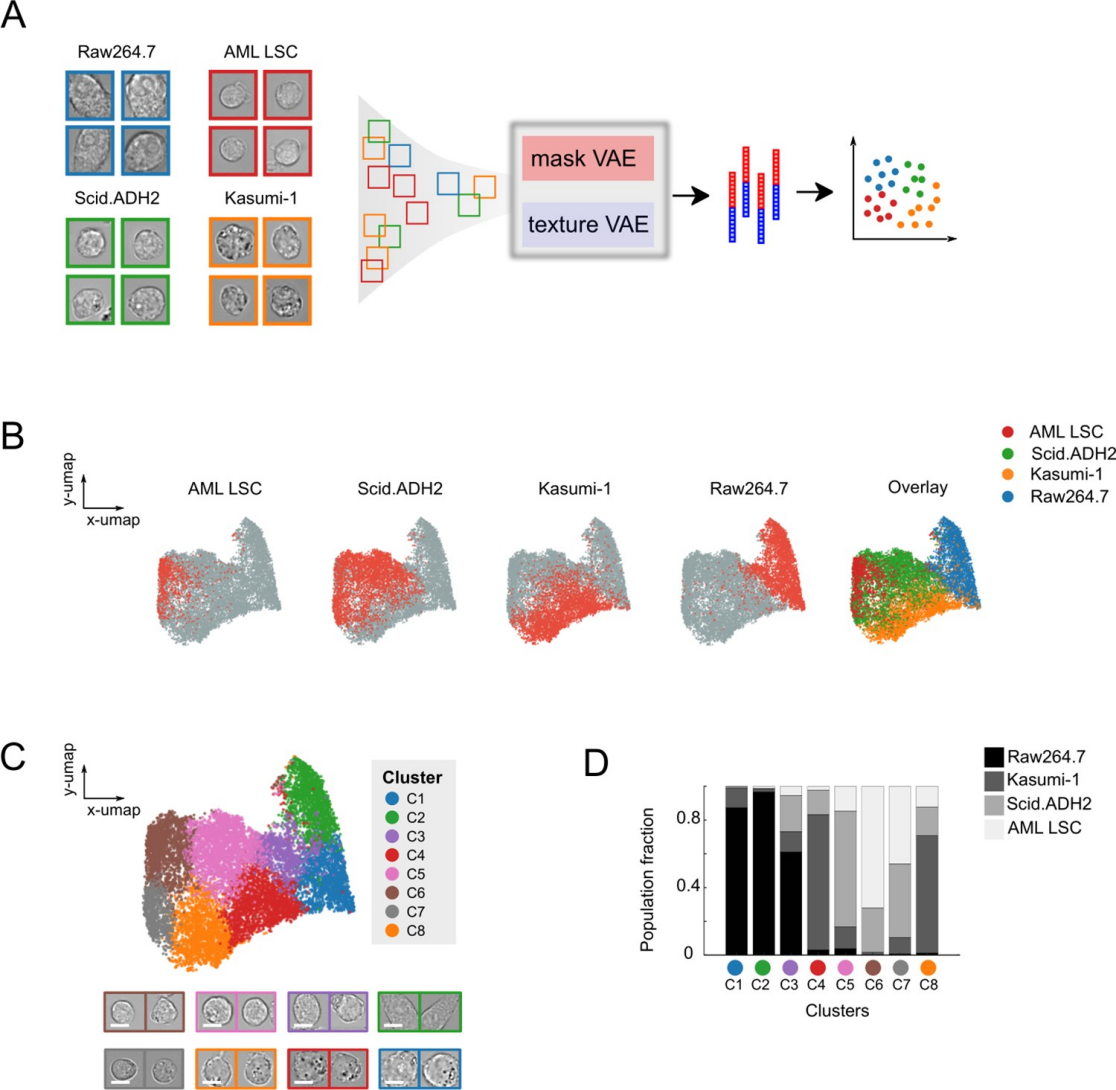
UPSIDE distinguishes morphologically-distinct blood cell types in a heterogeneous population. (A) Images of four different blood cell types were mixed together and passed through the UPSIDE workflow. Resultant shape and texture images were used to train concurrent VAEs. Output latent encodings were weighted relative to each other, concatenated, then projected onto a 2D plane using UMAP. (B) Dot plots show distribution of each cell type projected on 2D UMAP space made by UPSIDE. (C) 2D UMAP projection of the VAE-generated encodings that have been grouped into different morphological clusters using Louvain clustering algorithm. Representative brightfield cell crop images from the different clusters were listed. Scale bar represents 5 **μ**m. (D) Cell type fractional composition within each cluster. A fixed number of cells from each cell type were sampled, and the cluster-wised cell type composition was calculated from this pooled population.

We first tested UPSIDE’s ability to learn cell type-defining morphological features in a mixed dataset consisting of multiple blood cell types. To test the limits of UPSIDE’s ability, we chose four cell types that, despite having distinct size, shape and textural features, were similar in their gross morphologies (Figs 2A and S3A): a mouse T cell leukemia line (Scid.ADH2), a mouse macrophage cell line (Raw246.7), a human acute myeloid leukemia cell line (Kasumi-1), and primary patient-derived human acute myeloid leukemia stem cells (CD34^+^CD38^-^ AML LSC). Brightfield images from each cell population were captured, and cells were segmented using the neural network described above. Image crops of segmented cells were then mixed together and encoded into the latent space using UPSIDE’s VAE (S4A Fig). To quantify UPSIDE’s performance, we devised a cell type homogeneity score, which reflects how closely cells of the same type cluster together in their latent space (see *Methods* section*)*. We ran the VAE for this dataset for different values of Ω, to optimize the relative weights of the learned mask and texture encodings to achieve a maximal mean homogeneity score across the four cell types (S5A and S5B Fig and *Methods* section). This parameter specifies the relative importance of shape and texture to phenotypic state identification, and thus provides the ability to adapt UPSIDE to analyses of cell types and questions that rely on different types of distinguishing features. To compare the performance of the VAE to other deep learning methods, we repeated this analysis with several alternative architectures such as a vanilla autoencoder (AE) [25], an adversarial autoencoder with latent dimension encoding trained to fit a normal distribution or mixed gaussian distribution [14] (1xAAE and 4xAAE, respectively), and the ClusterGAN architecture [26] (ClusGAN) (see *Methods* *section*).

The VAE outperformed these other approaches, generating approximately 6% higher homogeneity scores compared to the adversarial autoencoders, 9% higher compared to PCA, and 26% higher than the ClusterGAN architecture (S5C Fig). Adversarial autoencoders performed better than the vanilla encoder though worse than the VAE, possibly because of difficulties in training the discriminator to fit the latent encoding to the desired distribution perfectly. Surprisingly, the ClusGAN architecture performed the worst, likely due to an inability to consistently generate direct, regularized encoded representations. Despite its superior performance compared to other architectures, the VAE achieved a maximal homogeneity score that was still significantly less than unity (~0.7, S5C Fig), indicating some degree of cell type mixing in latent space after learning. This was not unexpected given that some cell types – particularly AML LSCs and SCID.adh2C2 cells – appear visually similar or even indistinguishable in some cases, and cannot be separated by morphological characteristics observed from brightfield images alone. Nonetheless, these comparisons suggest that the VAE architecture is particularly well suited for learning morphological features for cell type discrimination.

To further visualize and analyze the representation of cells in latent space, we projected the encodings from the VAE into two dimensions using the UMAP algorithm [24] (Fig 2B). From the UMAP projection, we found that the cell types largely segregated into distinct regions in this two-dimensional space (Fig 2B). Raw264.7 macrophages occupied a region that was largely distinct from regions occupied by other three cell types, reflecting their markedly different size and shape distribution. The three other cell types occupied partially overlapping regions, reflecting greater similarities in morphology among these cells (S3A Fig). Interestingly, primary human AML stem cells (identified by their CD34^+^CD38^-^ surface marker phenotype) overlapped parts of the Scid.ADH2 region, suggesting some of Scid.ADH2 cells look quite similar to their AML counterparts. Despite these overlaps, there are substantial areas in the two-dimensional space occupied by these regions containing only one cell type, indicating the presence of morphological features that distinguish each of these three cell types from another and allow them to be identified in mixed populations.

To understand the morphological features driving cell type separation in this latent space, we clustered cell representations in the latent space using the Louvain method, then visualized cells and the morphological attributes that defined each cluster. Eight clusters were identified, with each enriched for different cell types (Figs 2C, 2D and S3B). Clusters C1-3 were highly enriched for Raw264.7 macrophages, phagocytic cells that are larger than their progenitor cells. Clusters C4 and C8 were highly enriched for Kasumi-1 cells, circular profile cells that contain dark granules, a unique distinguishing, observable feature of these cells. Cluster C5 was enriched for Scid.ADH2 cells, which are also circular, but lacked granules. Clusters C6 and C7 were enriched for both LSCs and Scid.ADH2 cells, both of which were small and lacked granules. Cells in Cluster C7 have darker interiors and less well-defined cell boundaries compared to Cluster C6 cells, indicating that they are flatter and may be more substrate-adherent. The morphological differences within these clusters indicate the existence of distinct morphological sub-states within individual cell types.

**Fig 3:**
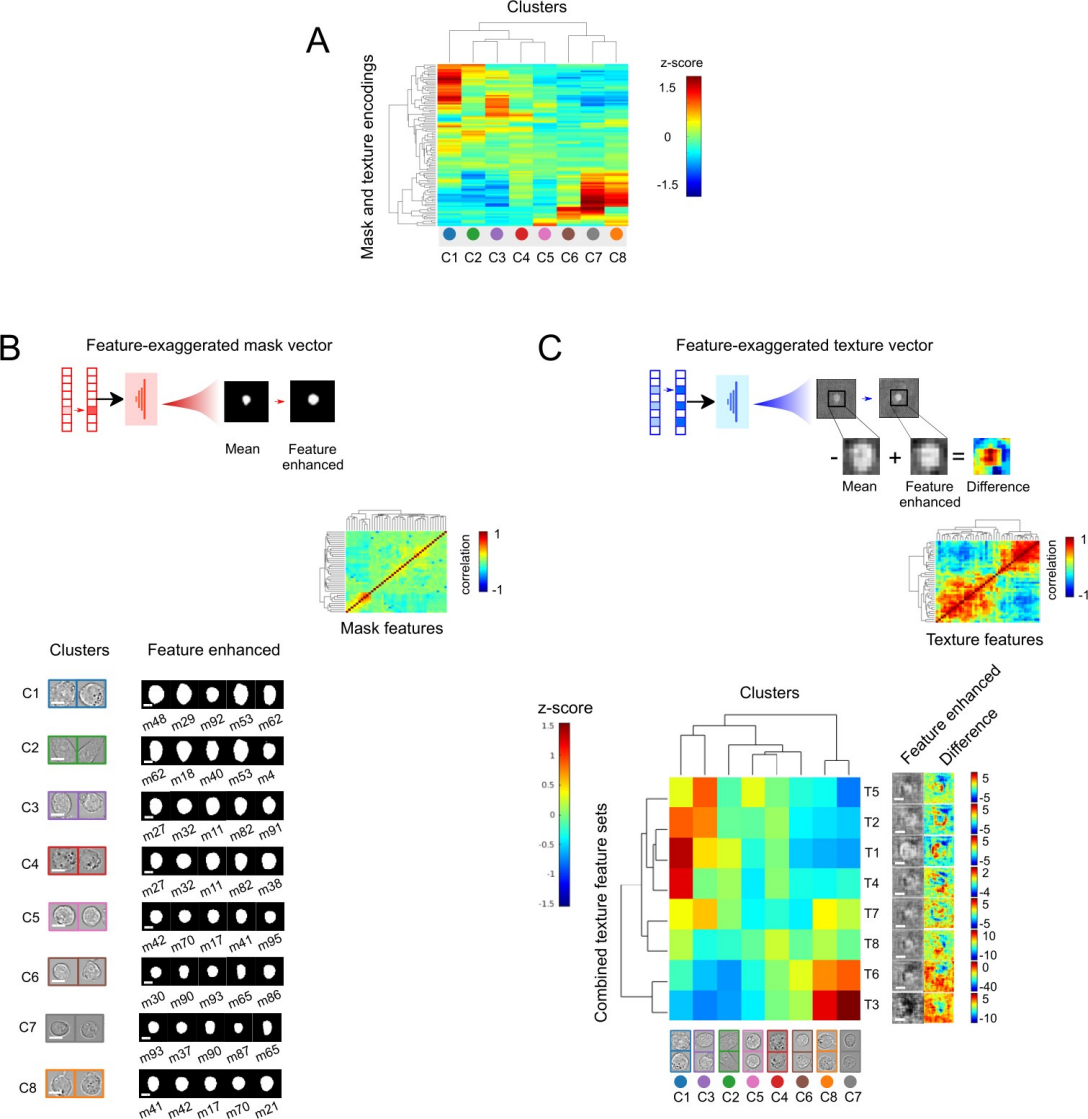
Cell-type specific morphological features can be interpreted by decoding the latent space cell representation. (A) Clustergram of average z-scores for latent shape and texture features for different cell clusters (see *Methods* section for how z-scores values were calculated). (B) Five mask features with highest z-scores for each morphological cluster are decoded and visualized. Clustergram shows matrix of correlation coefficients for forty mask features having the highest standard variation in the dataset. Scale bar represents 5 **μ**m. (C) Individual texture features were clustered into eight groups (T1-T8) according to their correlation with each other from the clustergram of texture features. Each group was decoded into brightfield difference images for interpretation (see Methods). Scale bar represents 5 **μ**m.

To interpret and visualize the morphological features that separate cells into distinct groups in latent space, we performed hierarchical clustering on the averaged latent space representation for cells from different clusters (Fig 3A). This analysis revealed that each morphological cluster of cells is associated with a specific set of latent features, with magnitudes that are higher than population average. To decode these latent features, we transformed them back into synthetic images in visual space (Fig 3B and 3C, top). First, we generated a mean mask or texture vector by averaging over all cells in the dataset. From these mean vectors, we then selectively increased the magnitudes of the feature (or groups of features) of interest to generate a new vector. Using the VAE decoder module, we then transformed the feature-exaggerated vector and the mean vector into synthetic images for interpretation.

We first examined the synthetic decoded images from the five most enriched mask features for each morphology-defined cluster (Fig 3B, bottom). Clusters C1-4 contained large cells with large, round profiles. As expected, cluster C2 contained elongated cells with numerous elongated mask features. Clusters C5-8, in contrast, contained smaller cells enriched in features representing small, round profile cell shapes. These mask features are in general agreement with sizes and shapes for cells found within individual clusters (Fig 2C).

Unlike mask latent features, individual texture features in the latent space were not readily interpretable for this dataset. However, because magnitudes of the projections along individual texture dimensions correlated strongly with each other in distinctive groups (Fig 3C insets) – in contrast to those for individual mask dimensions (Fig 3B, inset) – observable texture features in the image space might be encoded not by individual latent features, but by groups of correlated latent features. Therefore, to visualize these observable features, we generated feature-dominated vectors by concurrently increasing groups of correlated latent features. We also calculated images representing the difference between feature-exaggerated decoded images compared to the mean texture decoded image for better visualization. From these synthetic difference images, we observed two overall texture pattern groups: one with darker cell interiors (T3, T6), indicative of a flatter morphology; and the other with lighter cell edges (T1, T2, T4, T5), indicative of a less flattened morphology (Fig 3C). The darker cell interior feature group is strongly enriched in Clusters C7 and C8, while the lighter cell edge group is significantly present in Clusters C1 and C3. Clusters C2, C4, C5, and C6 appear to have moderate enrichment in all these groups.

Taken together, these results reveal how UPSIDE separates cells into distinct morphological clusters by their size, shape and distinct textural features. This ability can be seen readily in Clusters C3 and C4, where cells of similar size and profile can be discriminated based on their cell edge texture features. Cells with similar textural features can also be discriminated using other features, e.g., Custers C7 and C8 are both enriched with dark cell interior textures, but differ in size with Cluster C7 cells larger on average than those in Cluster C8. These results demonstrate that UPSIDE can generate meaningful learned morphological features in an unsupervised manner, and these features can be effectively decoded into images to aid interpretability. This ability allows UPSIDE to extract valuable morphological properties by simply observing cells over time without prior manipulation or human annotations.

### UPSIDE uncovers morphologically distinct cell states in patient-derived leukemic cells

LSCs play critical roles in AML disease propagation and drug resistance [27,28]. LSC and other AML cell subpopulations are typically identified and characterized by a combination of cell staining for granule content and cell surface markers as well as by their gene expression signatures [29,30]. All of these classification approaches can be further extended by transmitted light imaging and analyses to provide complementary information about leukemic cell types and states that is not readily obtainable through more conventional classification approaches. In particular, live cell movies that resolve phenotypic states over time and in response to pharmacological treatment could provide unique insights into cellular heterogeneity and responses that could better inform therapeutic decision-making.

**Fig 4:**
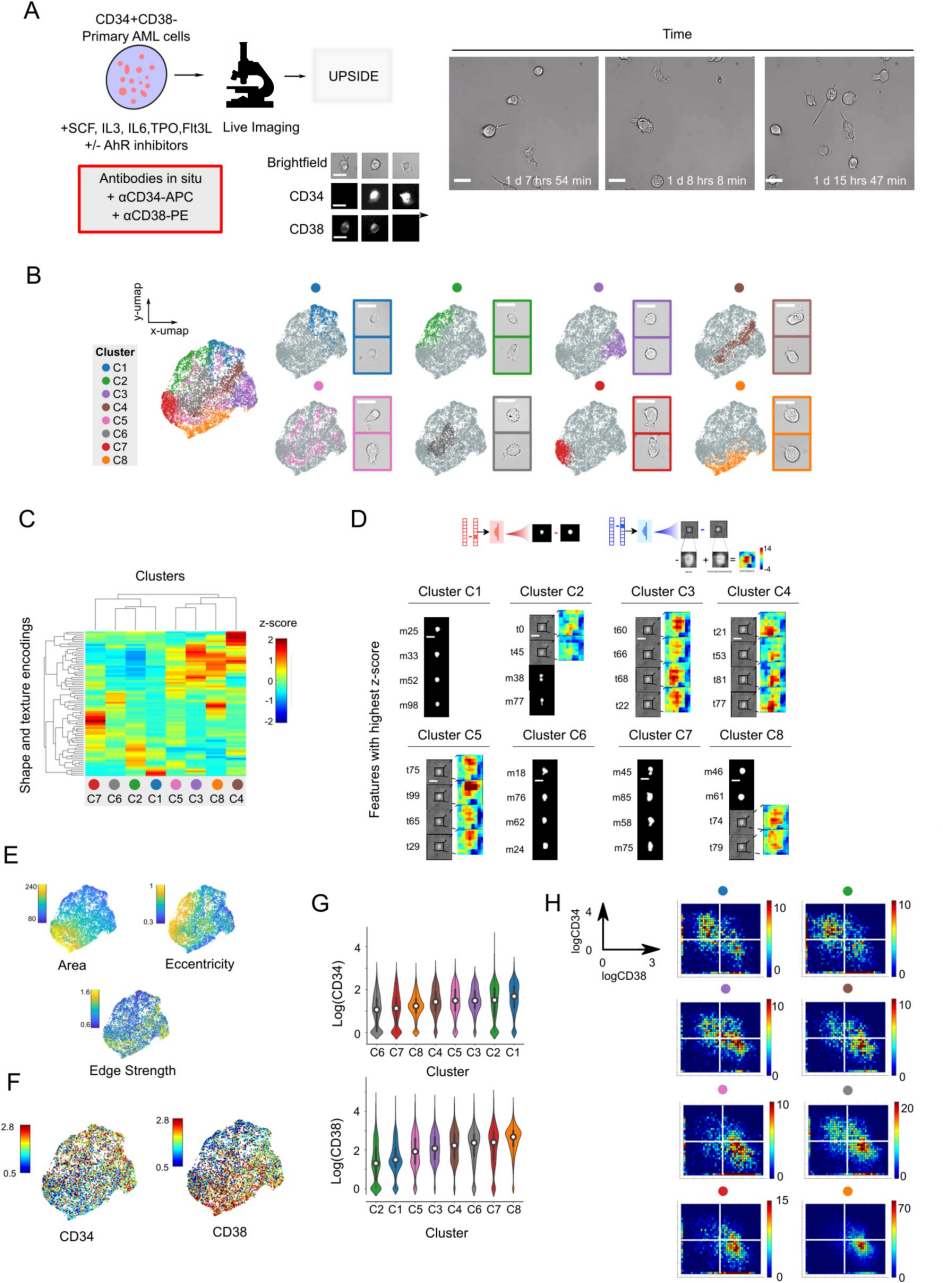
UPSIDE identifies stem cell-associated morphological states from patient-derived AML leukemic cells. (A) LSCs (CD34^+^CD38^-^) from an acute myeloid leukemia patient were cultured in cytokines with or without AhR inhibitors (UM729 and StemRegnin1) filmed for ~5 days (left). Brightfield images were captured once every 3-5 minutes. αCD34-APC and αCD38-PE antibodies were added in situ, and fluorescent images were captured once every hour (top right). Still frames show representative time lapse images of AML cells (bottom right). Scale bar represents 10 **μ**m. (B) UMAP 2D projection of the UPSIDE generated latent space cell representations. Individual morphological clusters were identified using the Louvain Clustering algorithm, then grouped manually based on their proximity to each other in the latent space (See S6B Fig). Representative cell images from each cluster were also shown. Scale bar represents 10 **μ**m. (C) Clustergram shows Z-scores of the latent mask and texture encodings for each morphological cluster. (D) Decoded images of the four most enriched features for each morphological state. Texture features were visualized using difference maps that were zoomed in around the decoded cells. (E) Area, eccentricity, and edge strength for each cell were calculated and mapped to the UMAP latent space representation. (F) CD34 and CD38 levels were mapped onto the UMAP. (G) Violin plots show distributions of CD34 and CD38 expression levels in different morphological clusters (left). (H) Histograms showing log CD34 levels against CD38 levels for each morphological cluster (right).

Towards this end, we employed UPSIDE to profile primary human LSCs cultured under cytokine conditions promoting expansion and differentiation, and filmed using brightfield imaging (Fig 4A, left). We directly isolated CD34^+^CD38^-^ leukemic stem cells from an adult AML patient [27,31,32]. This population is associated with chemotherapy resistance and persistent disease^32^, and likely serves as a reservoir of drug resistant cells that fuel relapse after chemotherapy treatment. To profile the self-renewal and differentiation dynamics of these sorted cells, we then cultured LSCs with either IL-6 and thrombopoietin (TPO) to induce differentiation, or with Aryl hydrocarbon receptor inhibitors (AhRi) UM729 and SR1 to maintain stemness and suppress differentiation [33–35]. We then filmed these cells in the brightfield channel for ~4 days at high temporal resolution (3 minute intervals, Fig 4A). To determine the association between observed cell morphological states, stemness and differentiation, we also added fluorescently-labeled anti-CD34 and anti-CD38 antibodies in culture, and took fluorescent images every hour to follow expression of these markers in imaged cells (Fig 4A, top right). Such *in situ* antibody labeling allows real-time visualization of cell surface marker protein expression with minimal effects on cell viability [36]. UPSIDE is well-suited to facilitate these types of time course analyses and image-based profiling: the use of brightfield imaging obviates the need for genetic engineering of fluorescent reporters to allow a wider range of analyses to be performed on primary patient-derived cell samples. It also minimizes cellular phototoxicity, thus enabling long-term cell observation at high temporal resolution.

Resultant timelapse images revealed considerable heterogeneity in the morphologies of observed cells, with these cells differing in their sizes, shapes and textures, as observed by their contrast from transmitted light images (Fig 4A, right; S1 Movie). To better understand this morphological heterogeneity, we fed these images into the UPSIDE pipeline. To minimize batch effects, we analyzed cells from all time points both with and without AhRi treatment in a single encoding run. We segmented cells, then encoded using UPSIDE’s separate shape and texture VAEs (S4B Fig). We then clustered representations of cells in latent space using the Louvain method (S6 Fig). Based on proximity in the latent space, we further combined some of the clusters we obtained into larger clusters. To visualize these clusters in two-dimensional space, we then projected these latent representations onto a two-dimensional plane using the uniform manifold approximation and projection algorithm (UMAP). This projection revealed the locations of the discrete clusters, along with their overlap regions. In this two-dimensional visualization, some cell clusters showed considerable boundary overlap with others, reflecting the continuous nature of the latent features encoded by the VAE. Additionally, imaged cells occupied all clusters both with or without AhRi treatment (S7 Fig), though the distribution of cells in these populations varied between the two conditions, likely reflecting the result of AhRi treatment on the cellular state.

In order to gain insight into the features that drive the separation of the cell encodings into distinct clusters, we performed hierarchical clustering on averaged cell encodings from each group (Fig 4C), then decoded the specific mask or textural features with the highest z-scores in each group to generate feature-exaggerated synthetic images, as described above (Fig 3B and 3C). These synthetic images highlight significant morphological features that display coherence across cells within a cluster, but differ between cells in different clusters (Figs 4D and S7 and S8). Examples of coherent morphological features include size, with some having smaller cells (Clusters C1,C2) and others having larger cells (Clusters C6,C7,C8); cellular elongation or eccentricity, with some clusters displaying rounder cell profiles (Clusters C1,C8) and others have more elongated cells (Clusters C2 and C7); and the presence of finer morphological features, such as cytoplasmic protrusions from the cell body (Clusters C6 and C7). Another important morphological feature was the degree of contrast at the cell edge observed from brightfield images, an indication of the degree of cell flattening on its culture surface. Some clusters had cells with weak contrast at edges (C1, C2 and C6), indicative of a flattened morphology, whereas others had cells with stronger edge contrast (C3, C8), consistent with a rounder, less-flattened appearance (S7 Fig). Together, these results reveal the distinct, defining morphological features of cells that are found in different morphological states.

To verify that these differences in decoded features indeed reflect systematic morphological differences between cells in different clusters, we calculated cell area, eccentricity, and edge strength – defined by the maximum value of the cell’s gradient image – and then plotted these quantities onto the 2D projection of the latent space (Fig 4E). Indeed, regions occupied by the different cell clusters had area, eccentricity, and edge strength values consistent with what was generally observed in the decoded cell images: Clusters C1-3 resided in the region with small cell areas, whereas Clusters C6, C7 and C8 resided in the region with larger cell areas. Elongated cells in Clusters C2 and C7 resided in regions with high eccentricity, whereas cells with darker cell edges in Clusters C3 and C4 resided in regions with high edge strength. Together, this analysis shows that UPSIDE can elucidate defining shape and textural features of cells that can vary across a population.

### Distinct morphological states are associated with different degrees of stemness

Cells in the different morphological states identified above may exhibit different degrees of AML cell stemness or differentiation. To test this idea, we investigated the relationship between these morphological states and the expression levels of CD34 and CD38, which together specify the stemness of these cells. To do so, quantified CD34 and CD38 expression levels for each cell, and mapped them onto the 2D projection of AML cell’s learned latent dimensions (Fig 4F and 4H). We also generated 2D heatmaps of these markers on a log-log axis, both for cells within individual clusters and for all cells (Fig 4H, right; and S8B Fig, left, respectively). From this analysis, we identified morphological clusters enriched for cells in either a stem cell state (CD34^+^CD38^-^) or a more mature state (CD34^-^CD38^+^). Specifically, morphological clusters C1 and C2 were enriched for the stem cell population (CD34^+^CD38^-^). Cells in these clusters differed in their roundness, but were uniformly small, consistent with the quiescent nature of the stem cells. Remarkably, these cells were also flat on the 2D culture surface possibly reflective of the high expression levels of cell adhesion proteins in hematopoietic stem cells [37]. In contrast, morphological clusters C6, C7 and C8, were most enriched for the mature cell population (CD34^-^CD38^+^). These cells were uniformly larger, consistent with the larger size of more differentiated cells, but had varying shapes and degrees of flatness. This diversity in shape and substrate adherence likely reflects the multiple differentiated cell types that can emerge under these *in vitro* differentiation conditions. Consistently, when we gated cells in either a stem state (CD34^+^CD38^-^) or a mature state (CD34^-^CD38^+^) and analyzed their morphological cluster composition, we found that the more immature (CD34^+^CD38^-^) cell population had higher C1 and C2 cell fraction compared to the mature state (CD34^-^CD38^+^), but a lower C6, C7 and C8 cell fraction (S8B Fig, right). Together, this analysis reveals distinct morphological features associated with different degrees of stemness or maturity in AML.

### Population dynamics of cell morphological states

**Fig 5:**
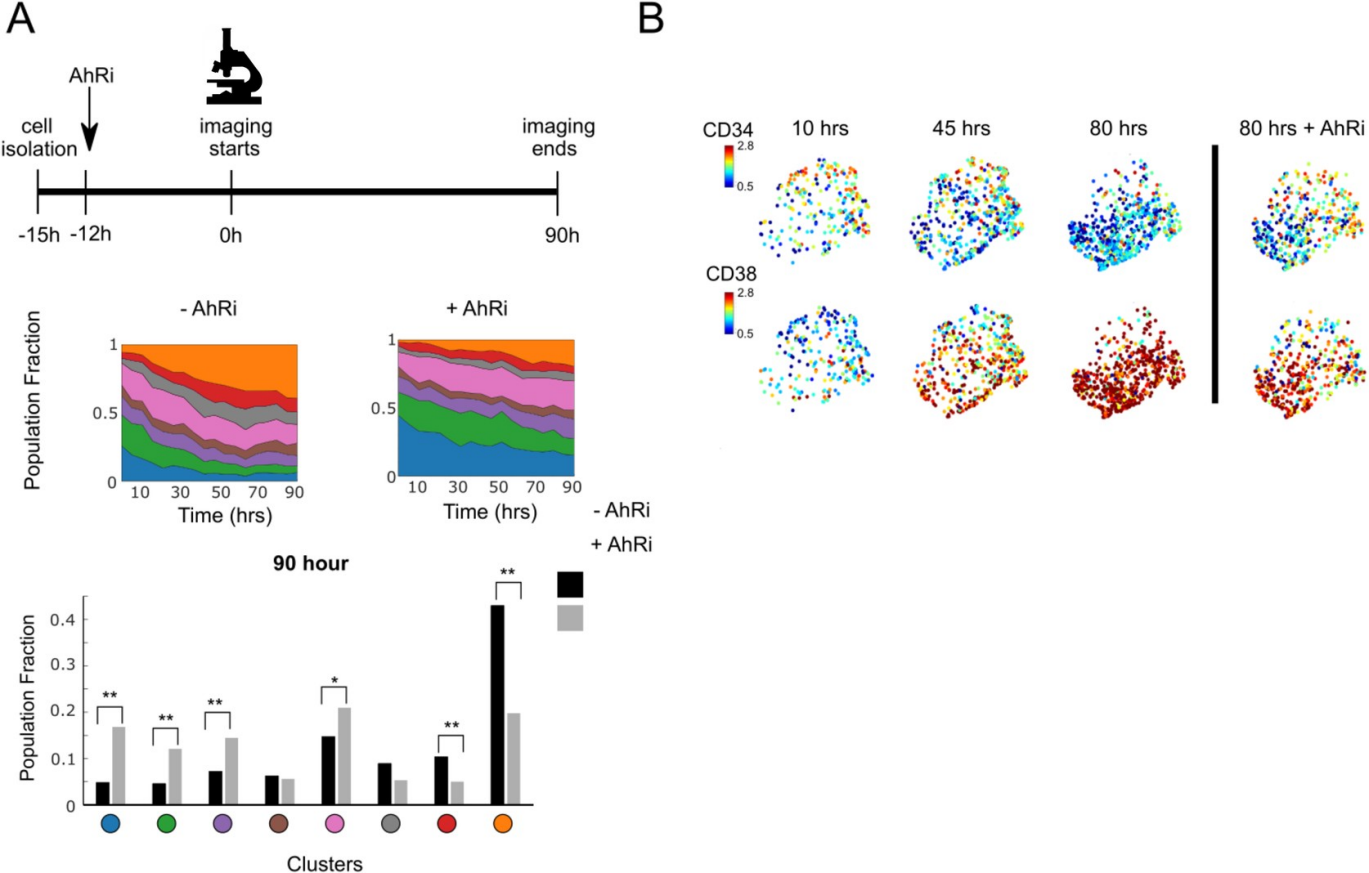
Population dynamics of identified morphological states. (A) Population fraction dynamics over time for each morphological cluster with (right) or without (left) AhR inhibitors (top). Population fraction contribution of each cluster at the last time point of the culture (bottom). Comparisons of the population fraction with and without AhR inhibitor were performed using the Chi-Square test for the dependency between the AhRi treatment and a cell’s cluster identity. **: p < 0.001, *: p <0.05 (B) UMAP showing CD34 and CD38 expression levels at different time points, in the presence or absence of AhRi.

To gain insight into the population dynamics of cells in different morphological states, we examined how the numbers of cells in different clusters evolve over time, both with and without suppressing differentiation with AhR inhibitors (AhRi; Figs 5A and S9A). In the absence of AhRi, cell clusters enriched for stem cells states (Clusters C1 and C2) are progressively depleted, while those enriched for more mature cells (Cluster C8) expand, consistent with the maturation of LSCs into more differentiated cells over time. As expected, AhRi treatment increases the sizes of the clusters enriched for stem cells (C1 and C2) relative to untreated conditions, while decreasing the sizes of the clusters enriched for mature cells (Cluster 8). This reflects the known effects of AhRi in maintaining stem cell self-renewal. The fractions of cells in intermediate morphological states (Clusters C3-7) remain largely unchanged, regardless of the presence of AhRi, suggesting that AhR inhibition may affect intermediate state transitions without driving specific outcomes. At the same time, CD34 levels decrease whereas CD38 levels increased over time, with both these changes becoming less pronounced with the addition of AhRi (Figs 5B and S9B). Together, these results provide insights into the population dynamics of LSC self-renewal and differentiation, and how these dynamics are affected by pharmacological compounds that modulate self-renewal.

Parallel experiments were used to further explore morphological changes that reflect LSCs maturation. We cultured LSCs (CD34^+^CD38^-^) in parallel with live imaging experiments, and analyzed them after three days for the expression of CD34, CD38, and CD123, another common LSC marker [38] (S9C Fig). Compared to untreated samples, cells treated with AhRi show higher expression of CD34 and CD123. On the other hand, CD38 expression magnitude was higher in the untreated sample, indicating greater differentiation in this population. Of note, a population of cells expressed both CD38 and CD34; this result indicates that expression of these markers may not be mutually exclusive. Parallel live imaging experiments of AhRi-treated cells showed slower expansion of large round cell morphology clusters compared to their untreated counterparts (Figs 5A and S9A). Together, these results demonstrate that distinct cell morphological states identified using UPSIDE indeed reflect leukemic cells in different states of maturation.

### Inference of morphological state transitions by cell linkage analysis

**Fig 6:**
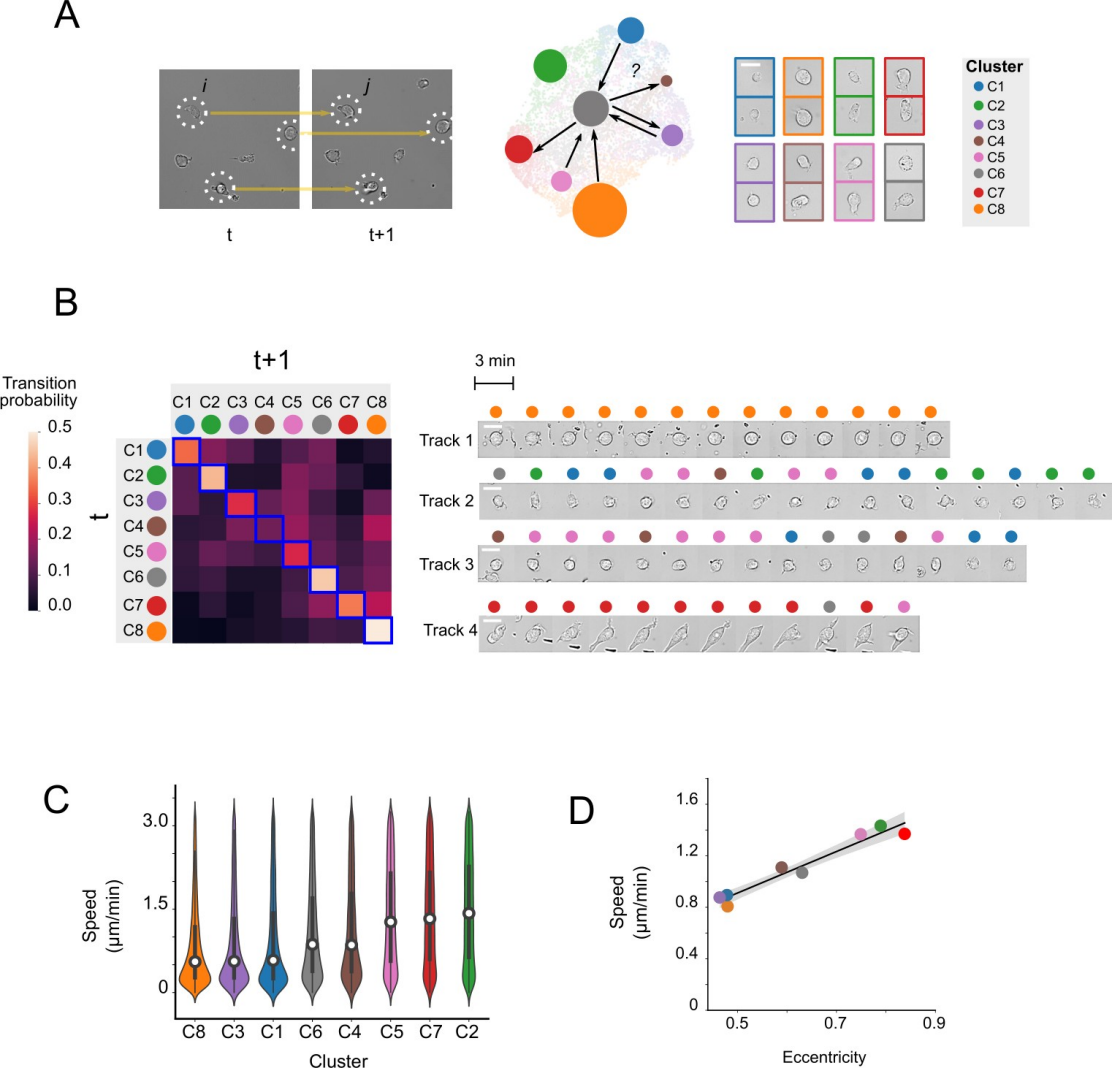
Calculation of morphological state transition probabilities by cell linkage analysis. (A) Cell pairs found in proximity across on successive time points were linked (left). Cell linkages, along with assigned morphological states of linked cells, were used to calculate transition probabilities between all states. (B) Heatmap shows transition probability matrix between all morphological clusters (left); image montage shows representative cell tracks identified from the culture (right). Scale bar represents 10 **μ**m. (C) Distribution of cellular velocity for linked cells for each morphological cluster. (D) Plot shows mean cell velocity against mean cell eccentricity for each morphological cluster.

The high temporal resolution of the brightfield movie analyzed above enables tracking of individual cells from frame-to-frame and, in conjunction with UPSIDE, inference of the rates at which leukemic cells transition between different morphological states. Notably, this analysis enables inference of rates without generation of long cell trajectories, which is particular challenging due to rapid cell motility of AML cells under investigation. Here, we develop an analysis routine to automatically infer transition rates from brightfield movies. In particular, this UPSIDE-enabled analysis obviates the need for generating individual cell tracks, which are typically error-prone and require considerable manual intervention. We paired cells from adjacent frames together, based on their close proximity. We then identified the morphological states of linked cell pairs using the VAE above (see *Methods* section), and calculated state transition probabilities based on the frequencies of linked cell pairs with specific initial and final morphological states (Fig 6A). By repeating this calculation over all possible pairs of morphological states, we obtained a matrix, describing the transition probabilities between different morphological states (Fig 6B, left).

This analysis revealed that cells largely transition between different morphological states in a highly dynamic and reversible manner, but exhibit state transition preferences that reflect underlying longer-term differentiation trends. Transition probabilities generally range between ~0.05 to 0.5 (/3 minute frame) (S1 Table), implying transition timescales of tens of minutes or less. However, these probabilities are not uniform, but are higher for specific transitions, such that the numbers of cells in different states changed progressively over time. Specifically, cells in the most immature states (C1 and C2), as defined by CD34 and CD38 expression (see above), tend to either inter-convert within these states, or switch preferentially to some of the more intermediate states (C5 and C6). In contrast, cells in the more intermediate states (C3, C4 and C5) tend to switch to one of the more mature states (C6, C7 and C8), though they also transition back into the an immature state with lower probability (e.g. C3 → C1; C5 → C2). Interestingly, intermediate states appear to inter-convert in a more dynamic manner, such that probabilities of maintaining the same state over time were lower for these intermediate states than they were for either the immature (C1, and C2) or more mature (C6, C7 and C8) states. Finally, cells having the most mature states (C6, C7 and C8) tend to stay within these states, and more stably maintained these states compared to other cell populations. A similar maturation trajectory was observed when transitions were visualized with averaged vector fields on a UMAP projection of the latent space (S10C Fig). These vector fields emanated from the most immature states (C1 and C2), flowed through the intermediate states (C3-C5), then converged upon the most mature states (C6-C8). Moreover, the magnitude of these vector flows are higher within the immature and intermediate states (e.g. C1, C3 and C4) but lower for the mature states (C6 and C8), consistent with the stability of these states observed in the transition matrix. Differences in trajectory stability were also directly observed when following single cells; mature cells (those starting in state C7 and C8) tend to remain in the same state (Fig 6B right, tracks 1 and 4), whereas more immature cells (C1, C3 and C5) are highly dynamic, switching from one state to another rapidly between successive frames (Fig 6B, tracks 2 and 3). Together, these data indicate that cells transition rapidly and reversibly between alternate morphological states, but do so in a biased manner, such that they progress from more stem cell-associated states (C1 and C2) toward more mature cell states (C8).

What is the basis of these dynamic morphological state transitions? The AML cells we filmed move rapidly on the culture surface (S1 Movie), and polarize as they move; thus some of the observed morphological state transitions could reflect transitions from a stationary to a motile state. To test this hypothesis, we derived the instantaneous velocities of cells in different morphological states, by calculating the displacement between successive frames for each state (Fig 6C). From this analysis, we found that cells with elongated morphologies, such as those in states C2, C4, C5, and C7, show higher movement velocities compared to other states. Consistently, there was a strong correlation between instantaneous velocity and cell eccentricity, averaged over all cells in individual clusters (Fig 6D). Thus, though morphological transition probabilities are biased by differentiation, the transitions themselves involve the rapid switching between stationary and mobile states (Fig 6B, left, tracks 2-3).

If morphological state transition probabilities calculated above are biased by the directionality of cell differentiation, they would change when cells are subject to perturbations that modulate stem cell differentiation and self-renewal. To test whether this was the case, we repeated this state transition analysis for cells treated with AhR differentiation inhibitors (S10A Fig and S1 Table). In the presence of AhRi, there is a decrease in the transition probabilities into the more differentiated states (C6, C7 and C8), together with an increase in the transition probabilities into and amongst intermediate cell states (C3, C4 and C5). This suggests that AhRi blocks movement from a stem cell-associated morphological state to one associated with mature cells by stalling cells in an intermediate state. Consistently, single cell track analyses showed that cells often transition rapidly from an intermediate state (C5) to more mature state (C8) without AhRi, but stall at an intermediate state (C5) when AhRi is present (S10B Fig). Thus, these observations show that changes in cell differentiation dynamics can influence the probabilities at which cells transition between different morphological states.

## Discussion

In this study, we developed a deep learning platform, UPSIDE, for unsupervised exploration of dynamic cell morphological states in transmitted light movies. Using UPSIDE, we identified distinguishing cell morphological features and states in a heterogeneous collection of blood cell types. We found that the UPSIDE VAE learning architecture outperforms other comparable methods in recognizing unique morphological features within each cell type. We further demonstrated the utility of our method by uncovering morphological states in primary human AML patient-derived leukemic cells displaying different degrees of stemness, differentiation, and cellular mobility. Finally, UPSIDE addresses the issue of latent feature interpretability, one of the most challenging aspects of analyzing deep convolutional networks, to allow more intuitive insight into learned latent morphological features.

UPSIDE will enable analysis of dynamic responses of AML leukemic stem cells to chemotherapy at the single cell level. AML drug resistance poses a significant clinical challenge as the majority of patients eventually develop relapse disease. Growing evidence suggests leukemic stem cell populations in AML constitute the drug-tolerant sub-populations that survive drug treatment and eventually lead to relapse disease [28,39]. A number of studies have profiled the genomic and transcriptomic features of these stem cell populations at the single-cell level [40,41], however, single-cell profiling approaches that continuously monitor differentiation and drug resistance dynamics in single cells could yield additional information not readily obtainable from these snapshot measurements. To this end, a large scale screen of hundreds of drug treatment or regimen time courses can be captured via brightfield time lapse imaging [42,43]. UPSIDE can then be employed as an unbiased method to survey and specify important morphological features associated with therapeutic response, persistence or resistance as a function of cell types, cell states and treatment.

More generally, UPSIDE has a number of advantages that facilitate the unbiased exploration and discovery of dynamic cell phenotypes from microscopy movies of unlabeled cells. As UPSIDE does not require genetic labeling, identifying or tracking cells, it could readily be applied towards a wide variety of cell types, including primary cells and patient samples. Furthermore, the reduced phototoxicity of transmitted imaging allows for high temporal resolution imaging over long times, allowing dynamic transitions to be analyzed over a wide range of timescales. Finally, the unsupervised nature of UPSIDE makes it particularly suited for analyzing new systems without prior feature selection or knowledge of underlying biology, as the VAE architecture is capable of self-learning the distinguishing features. In the future, integration of UPSIDE with image-based cell sorting techniques (e.g. [44]) will allow for cells with defined morphological features to be isolated for downstream analysis, thus opening the door to interrogation of the molecular basis of distinct phenotypic states.

## Methods

### Ethics statement

Human blood and bone marrow samples were obtained by written informed consent on a protocol approved by the University of Washington/Fred Hutchinson Cancer Research Center Cancer Consortium Institutional Review Board. The samples were deidentified in the laboratory.

### Experimental techniques

#### Cell lines

Kasumi-1, Scid.ADH2, and RAW246.7 cell lines were cultured Eagle’s minimal essential medium (DMEM), phenol-red free containing 10% Fetal Bovine Serum (FBS), Penicillin-Streptomycin-Glutamine (Gibco 10378016) at 37°C and 5% CO_2_ (ThermoFisher) for 2 days before imaging. For 5 cell type imaging experiments, each cell line was imaged in a separate individual well in the same 96-well glass-bottomed plate. AML211 CD34^+^CD38^-^ subpopulation was cultured in ‘Differentiation Media Condition’ for 2 days before imaging at the same time with the cell lines.

#### Culture of patient-derived leukemic cells

Primary acute myeloid leukemia samples (AML211) were provided by the Pamela Becker lab. The study was conducted with approval of the Institutional Review Board, Fred Hutchinson Cancer Research Center. The samples were obtained from AML patients with written, signed informed consent.

Cryo-preserved AML cells were thawed in ‘Long Term Bone Marrow (LTBM) Media’ [Iscove’s Modified Dulbecco’s Medium (IMDM) with glutamine and HEPES (Mediatech. Inc, Manassas, VA), 15% Fetal Bovine Serum (HyClone, Logan, UT), 15% Horse Serum (VWR), 50 μM beta-mercaptoethanol (Sigma), 0.043% Monothiolglycerol (Sigma)] and washed twice to remove DMSO, then cultured in LTBM with 50U/ml DNase to break up and free live cells if chunks are present at 37°C and 5% CO_2_ for 1 hour. Cells were then cultured in LTBM with 10 ng/ml recombinant human Stem Cell factor (SCF) at 37°C for 2 days. For cell sorting and flow cytometry analysis, cells underwent Lymphocyte Separation Medium (Mediatech. Inc, Manassas, VA) to remove dead cells and were stained with CD34 (ThermoFisher 17-0349-42), CD38 (ThermoFisher 12-0388-41), and CD45 (VWR 10758-692) for flow cytometry analysis and sorting by FACS Aria (BD biosciences, San Jose, CA).

Sorted CD45^+^CD34^+^CD38^-^ subpopulation from AML211 samples were cultured in ‘Differentiation Media Condition’ (adapted from (Klco et al., 2013)) which comprises of Eagle’s minimal essential medium (DMEM), phenol-red free containing 10% FBS, Penicillin-Streptomycin-Glutamine (Gibco 10378016), 100 ng/ml Recombinant Murine SCF (Prepotech 250-03), 50 μM beta-mercaptoethanol (Sigma M6250), 10 ng/ml Recombinant Human IL-3 (Prepotech 200-03), 20 ng/ml Recombinant Human IL-6 (Prepotech 200-06), 10 ng/ml Recombinant Human TPO (Prepotech 300-18), 10 ng/ml Recombinant Human Flt3-Ligand (Prepotech 300-19), or ‘Maintenance Media Condition’ (adapted from (Pabst et al., 2014)) which comprises of minimal essential medium (DMEM), phenol-red free containing 10% FBS, Penicillin-Streptomycin-Glutamine (Gibco 10378016), 100 ng/ml Recombinant Murine SCF (Prepotech 250-03), 50 μM beta-mercaptoethanol (Sigma M6250), 20 ng/ml Recombinant Human IL-3 (Prepotech 200-03),), 50 ng/ml Recombinant Human Flt3-Ligand (Prepotech 300-19), 1 μM UM729 (STEMCELL Technologies 72332), and 500 nM StemRegenin-1 (STEMCELL Technologies 72342). Cells were cultured on treated polystyrene (Corning) of glass-bottomed (Mattek) 96-well culture plate coated overnight with 33.33 μg/ml Retronectin (Takara T202).

For imaging differentiation assay, CD34 Human Monoclonal Antibody (4H11), APC (eBioscience 17-0349-42) and CD38 Human Monoclonal Antibody (HB7), PE (eBioscience 12-0388-41) was spiked into the culture media. Cells were imaged every 3-5 min with brightfield and 60 min with fluorescent light for 4 days. We note that low levels of fluorescence signal were present in the image channels, thus contributing to a non-zero background level in these cells.

#### Image acquisition

Timelapse imaging was performed on Inverted Microscope Platform Leica DMi8 (Leica Microsystem). All image acquisitions were performed using 40X air objective. Fluorescent images were captured using Laser Diode Illuminator, LDI (89 North).

### Image analysis

UPSIDE computational pipeline is designed to analyze the morphological diversity of cells from timelapse brightfield images. The method consists of four main modules: 1) Label-free prediction, 2) Image segmentation, 3) Live cell classification, and 4) Unsupervised feature learning. The following section describes each of the modules in further details.

#### Label-free imaging and image segmentation

UPSIDE utilizes a label-free imaging method to identify cells from brightfield (BF) images. Here we adapted a U-net-based deep learning technique described by Ounkomol and coworkers [22] to predict fluorescent pictures of cytoplasm from the captured BF images. To complete this task, we analyzed ~10^3^ cells stained with CellTrace Violet Cell Proliferation dye (ThermoFisher C34557) to label their cytoplasm. This cell number was sufficient to achieve maximal performance in cell prediction, as determined by training runs with different cell numbers (S1C Fig). Training data was obtained by capturing approximately 300 – 400 BF images and their corresponding cytoplasm fluorescent images. This data was then used to train a model that predicts cellular cytoplasm. These two models were subsequently used to predict fluorescent images for the main timelapse brightfield image stacks. Object segmentation was performed on predicted cytoplasm images using ictrack software [20]. To demonstrate the performance of this software, we sampled ~1000 segmented objects by ictrack and manually evaluated its segmentation accuracy by comparing these segmented objects to the predicted fluorescent images as reference. Objects that are properly identified based on their predicted fluorescent signals are considered correctly segmented, whereas those that are over-segmented or under-segmented are considered incorrectly segmented. We note that a significant fraction of incorrectly-segmented objects (7%) correspond to dead cells or cell aggregates that are then removed in the subsequent live dead classification step (S2B Fig).

#### Live cell classification

Identified cell crops were then fed through a classifier to separate living cells from dead cells and other debris for analysis by UPSIDE. We performed this additional classification step as dead cells and other non-cell objects possess a variation in shape, size and texture (S2B Fig) that could potentially affect the encoding of live cell morphological features. These concerns notwithstanding, we found that the outcome of UPSIDE’s clustering was not significantly affected by the presence of a small percentage (7%) of dead cells (S2C and S2D Fig).

We built a convolutional neural network for this classification task (S11 Fig):

To obtain training data for this network, ~10000 brightfield cell crops were manually annotated as ‘Live’ or ‘Dead.’ The network were trained for approximately 10,000 steps, and cross entropy loss were calculated a Adam optimizer^46^ were used for weights and biases learning:

$$CEloss=\sum_{i=1}^{j}\left[f(X)log(y)+(1-f(X))log(1-y\right)]$$ (1)

Where *f*(*X*) is the predicted class of a given cell crop *X* and *y* is its correct label. The remainder of the identified cell crops were then fed to the trained classification model. Crops classified as ‘Dead’ were discarded, and ‘Live’ crops were used for further analysis.

#### Unsupervised feature learning

Morphological feature learning in UPSIDE relies on the variational autoencoder architecture (VAE) [21] to perform feature extraction. Two information pieces were used to train the VAE: 1) The overall shape of the cell and 2) The cellular texture inside the boundary mask. Predicted CellTrace violet signal of the cell was used to generate the cell shape crop. The following image preprocessing steps were performed to minimize trivial variations between cell crops:

- Object re-centering
- Object rotational orientation to 90^o^. All cell crop images are then rescaled accordingly to eliminate image’s dimension inflation due to rotation.
- Object’s vertical and horizontal pixel density reorientation to top and right, respectively

To obtain texture representation, brightfield pixel values distribution inside the cell’s mask was scale adjusted to zero mean and unit variation. They are then scaled linearly to be between 0 and 1 to facilitate learning with VAE. All pixel values outside the boundary were set to 0.5.

Preprocessed image crops for shape and texture were used to train two separate VAEs. The overall architecture is as described below (S12 Fig).

The loss function for the VAE is a weighted combination between reconstruction loss and Kullback-Leibler Divergence loss:

L=A[γ·MSE+(1−γ)·KLD] (2)

where A is a constant, and γ varies between 0 and 1. As previously discussed [21], optimal values for γ are determined by testing different values, and manually examining resultant decoded images to determine the accuracy and diversity of output images with respect to its original image. Additionally,

$$MSE={\left||X-F(z,X)|\right|}^{2}$$ (3)

$$KLD=-\frac{1}{2}\sum_{i=1}^{J}\left(1+log(\sigma^{2})-\mu^{2}-\sigma^{2}\right)$$ (4)

VAE for cell shape feature extraction was first trained for ~100000 steps while VAE for texture feature extraction was first trained for ~200000 steps. Trained weights and biases for the cell shape extraction were then used to encode all cell crops obtained from the movie into 100-element vectors. These vectors were projected onto a 2D plane using UMAP [24]. Cell crops with defective shapes are gated out using the cytometry2 function in ictrack. The remaining crops were then used to train VAE for cell shapes and texture separately. Afterward, cell crops were encoded into 100-element shape vectors and 100-element texture vectors. Each cell crop’s latent vector is represented by a weighted concatenation between the shape and the texture contributions:

$$z=concatenate(w\cdot z_{shape},(1-w)\cdot z_{texture})$$ (5)

Encoded latent dimension of cell crops are then clustered using Louvain clustering algorithm.

To generate synthetic images, encoded cell barcodes and arithmetic variations were treated as z and fed directly into the decoder.

#### Comparable deep learning architectures

In addition to utilizing the Variational Auto Encoder architecture to learn the latent dimensions in our imaging datasets, we tested a few other deep learning architectures to compare their performances with our current approach:

*Vanilla Auto Encoder (AE)* [25]

In this architecture, each processed shape or texture is fed through a series of convolutional layers and fully connected neural network layers to generate a latent vector with a dimension of 100. The organization of the neural network layers are as follows (S13 Fig).

The loss function for the AE is:

$$L={\left||X-F(z,X)|\right|}^{2}$$ (6)

AE for cell shape feature extraction was first trained for ~100000 steps while AE for texture feature extraction was first trained for ~200000 steps. Each cell crop’s latent vector is represented by a weighted concatenation between the shape and the texture contributions:

$$z=concatenate(w\cdot z_{shape},(1-w)\cdot z_{texture})$$ (7)

*Adversarial Auto Encoder (AAE)* [14]

In this architecture, each processed shape or texture is fed through a series of convolutional layers and fully connected neural network layers to generate a latent vector with a dimension of 100. The latent dimension was then regularized using a discriminator that forces the dimension space into a unit gaussian distribution (1x AAE) or four mixed gaussian distributions (4x AAE). The organization of the neural network layers are as follows (S14 Fig).

The loss functions for the VAE are:

$$L_{autoencoder}={\left||X-F(z,X)|\right|}^{2}$$ (8)

$$L_{discriminator}=-\frac{1}{N}\sum_{i=1}^{N}\left[log(z_{real})+log(1-z_{encoded})\right]$$ (9)

Where *z*_*real*_ is a 100 element vector sampled from a normal gaussian distribution (1X AAE) or a mixed 4-gaussian distribution with each gaussian’s mean to be -1, -0.5, 0.5, and 0.5 and standard deviation to be 1 (4X AAE).

AAE for cell shape feature extraction was first trained for ~100000 steps while AAE for texture feature extraction was first trained for ~200000 steps. Each cell crop’s latent vector is represented by a weighted concatenation between the shape and the texture contributions:

$$z=concatenate(w\cdot z_{shape},(1-w)\cdot z_{texture})$$ (10)

*ClusterGAN* [26]

This architecture carries an encoder that converts a generated image into a latent dimension which is then forced to match the same starting latent code that was originally used to make the image. This is a semi-supervised architecture where a specific number of classes needs to be predetermined beforehand. To convert this into an unsupervised method, we removed the class module, enabling the GAN to draw data from a normal distribution, without the one-hot class vector input. The neural network organizations for the generator, encoder, and the discriminator are as follows (S15 Fig).

Loss functions used for training were described previously [26]. We input the cell crops into the encoder module of ClusterGAN to generate the latent dimensions for the comparative analysis with other architectures.

Cell shape feature extraction was first trained for ~100000 steps while the texture feature extraction was first trained for ~200000 steps. Each cell crop’s latent vector is represented by a weighted concatenation between the shape and the texture contributions:

$$z=concatenate(w\cdot z_{shape},(1-w)\cdot z_{texture})$$ (11)

### Algorithms and quantitative analysis

#### Neighbor similarity scoring

The metric is formulated to estimate the degree of homogeneity of the grouping of each cell type in the encoding space of the four cell types. Specifically, the neighbor similarity score *H*_*C*_ for a given cell type *C* is defined as follows:

$$H_{c}=E_{c}[\frac{\sum_{i=1}^{N}n_{i}}{N}]$$ (12)

Where *E*(·) represents the expectation value, or the mean over all cells within a cell type *C*, and *N* specifies a predetermined number of nearest neighbor cells to a given cell. We note that varying values of N does not significantly affect the score *H*_*C*_ (S5D Fig). Furthermore, for each neighboring cell *i*, *n*_*i*_ = 0 if the identity of *i* is *C*, and *n*_*i*_ = 0 otherwise.

#### Latent dimension z-score calculation

The z-score *Z*_*f*,*c*_ of a particular feature *f* of cluster *C* is defined as the fold difference in standard deviation between the mean of the value of that feature in cluster *C* compared to that of the complete dataset:

$$Z_{f,C}=\frac{\mu_{f,c}-\mu_{f}}{\sigma_{f}}$$ (13)

Here, *μ*_*f*,*C*_ is the mean value of feature f over all cells in cluster *C*, and *μ*_*f*_, *σ*_*f*_ are the mean and standard deviation of feature *f* over the dataset.

#### Pairwise cell tracking algorithm

The pairwise cell tracking algorithm was built to ensure the validity of a given paired cell linkage from one frame to another. To achieve this goal, we established stringent requirements for a given cell pair to be considered ‘valid’. Specifically, the linking algorithm concerning all cells in frame t is as followed:

$$||\;For\;all\;cell\;i\in N_{t}:$$

$$||\;if\;d(i,j)>D_{0}\;for\;all\;i\in N\;and\;i\neq j:$$

$$||\;for\;all\;cell\;k\in N_{t+1}:$$

$$||\;if\;d(i,k)<D_{1}\;and\;d(i,k)=min(V_{i}^{t_{+1}}):$$

||linkiandktogether

Here, *N*_*t*_ represents a set of all detected cells in frame *t*; *d*(*a*,*b*) denotes the Euclidean distance between cells *a* and *b*, and $V_{a}^{t}$ represents a set of Euclidean distances between cell *a* in frame *t*−1 to all cells in frame *t*. *D*_0_ is the minimum distance between the given cell and other cells in the same frame, for this cell to be considered for linkage analysis. It can readily be set by calculating the average distance between all cells in the dataset and setting a value of D_0_ to be larger than this average value. The parameter *D*_1_ represents the maximum distance a potential paired cell can be away from the initial cell. It can be readily determined by manual inspection of cell movements: By manually inspecting the distance traveled between cells in the movie, the user can have an expectation of how far a cell can reasonably travel between adjacent timepoints.

#### Transition probability between cell clusters

In order to estimate the transitional dynamics between identified morphological clusters through time, we determine the probability for a cell *X* to transition from cluster *i* at time *t* to another cluster *j* out of a set of clusters *k*∈*C* at time *t*+1 as follows:

$$p(X_{t+1}=j|X_{t}=i)=\frac{f_{ij}}{\sum_{C}f_{ik}}$$ (14)

Where *f*_*ij*_ is the number of transitions from cluster *i* to cluster *j*.

## Supporting information

S1 Fig Robust label-free cell prediction using the UNET architecture. (A) Sample images from models trained on brightfield images from the cell type dataset (top) and the Acute Myeloid Leukemia dataset (bottom). Scale bar represents 20 **μ**m. (B) Pearson’s correlation coefficients measuring correlation between the ground truth fluorescence images and predicted synthetic images. Asterisk (*) represents the theoretical upper limit of the model’s performance for each dataset. Such a model would perfectly predict the fluorescent level of each cell but not be able to predict fluorescent noise that arises from the instrumentation. (see^21^ for detailed method). (C) Pearson’s correlation coefficients for label-free prediction using different cell numbers for training. (D) Segmentation performance of the ictrack software (left) and sample segmented objects along with their brightfield and predicted fluorescence images (right). Scale bar represents 5 **μ**m.
(TIF)

S2 Fig A convolutional cell classifier is trained to remove dead cells from the dataset. Brightfield crops of selected cells are classified as either ‘Live’ or ‘Dead’ using a convolutional classifier that is trained to recognize dead cells using a manually labeled dataset. (A) Representative cell crops classified as ‘Live’ or ‘Dead’. Scale bar represents 10 **μ**m. (B) Receiver operating characteristic (ROC) curve measuring the prediction performance of the trained classifier. AUC: Area under the curve. (C) Fraction of correctly classified live cells out of ~1000 segmented objects. (D) UMAP plots show clustering of latent encodings of a cell population (top), with 100% confirmed live cells, 97% confirmed live cells, and 100% confirmed live cells with an unlearned variational autoencoder, where weights of neuron layers had randomized weights. Sample cell images from each identified cluster are also shown (bottom).
(TIF)

S3 Fig Images of the four blood cell types analyzed by UPSIDE. (A) Representative images from the four blood cell types Raw264.7, Kasumi-1, Scid-ADH2 and AML LSCs. (B) Representative images from eight different morphological clusters identified by Louvain clustering of the UPSIDE-generated latent vectors from each cell type. Scale bar represents 5 **μ**m.
(TIF)

S4 Fig Concurrent training of shape (mask) and texture variational autoencoders for the cell type dataset. Reconstruction and Kulback-Leibler Divergence (KLD) losses of the models for the Cell Types Dataset (A) and the Acute Myeloid Leukemia Dataset (B).
(TIF)

S5 Fig Comparison of cell type homogeneity scores obtained using different data encoding methods. (A) The cell type homogeneity score, defined as the mean fraction of the *N* nearest neighboring cells of the same type as the cell of interest, averaged over all cells, measures how well different cell types are separated in latent space. (B) Mean nearest neighbor score (H) across 4 cell types obtained with different relative mask weight contribution for encodings generated by either VAE or PCA method. (C) Maximum nearest neighbor scores (H_max_) for VAE, PCA, and other alternative deep learning architectures. H_max_ is defined as the highest mean nearest neighbor score across all weight combinations of mask and texture contributions. VAE: Variational Autoencoder, 4x AAE: Adversarial Autoencoder with latent dimension trained to fit a 4 mixed gaussian distribution, 1x AAE: Adversarial Autoencoder with latent dimension trained to fit a normal distribution, Clus GAN: Cluster Generative Adversarial Autoencoder with the one hot encoding component module removed, PCA: Principal Component Analysis. (D) Cell type homogeneity scores for the VAE, calculated using different numbers of neighbors *N*. (E). UMAP projections showing ground truth (left) and predicted clustering for the VAE (right), for two cell types, AML leukemic stem cells (LSCs) and Raw264.7 macrophages.
(TIF)

S6 Fig Clustering of AML cell morphologies in latent space using the Louvain method. (A) 2D UMAP projection of learned mask and texture encodings from combined AML datasets. Each cell was colored based on the raw Louvain clustering result over all datasets. (B) Clustergram of the z-score from morphological groups defined by Louvain methods. Groups with closely related z-score patterns were combined into larger morphological clusters.
(TIF)

S7 Fig Images and UMAP projections of cells from each grouped morphological cluster. (A) Representative imags of cells in different morphological clusters. Scale bar represents 10 **μ**m. (B) 2D UMAP projections of latent space encodings from the combined AML dataset separated into + and - AhRi conditions.
(TIFF)

S8 Fig Decoding of the four most enriched mask and texture features for each morphological cluster in image space. **(A)** Decoded texture images are accompanied by unzoomed pixel difference maps. Scale bar represents 10 **μ**m. (B) Heatmap presenting the distribution of CD34 and CD38 expression in AML LSCs after 90hrs of culture (left). Fractional composition of each identified morphological cluster for the CD34^+^CD38^-^ and CD34^-^CD38^+^ populations.
(TIF)

S9 Fig Time evolution of morphological states and CD34 and CD38 levels in AML cells. (A) Population fractions of cells in different morphological states in the absence(left) or presence of AhRi (center). Population fractions for cells in each cluster at the last time point (right). Comparisons of end-point population fractions of different morphological states, both with and without AhRi treatment, were performed using the Chi-Square test. **: p < 0.001. (B) UMAP of cells from the indicated time points. Unless otherwise indicated, cells were not treated with AhRi. Colors represent CD34 and CD38 expression levels at different time points. (C) Flow cytometry analysis of CD34, CD38, and CD123 expression levels of patient-derived AML cells cultured for 80 hrs, taken without imaging.
(TIF)

S10 Fig Transition dynamics between morphological clusters for AML cells with or without AhR inhibitors. (A) Transition probability matrices between identified morphological states with and without AhRi, along with matrices showing the difference between these two conditions (right); two replicates are shown (top and bottom). (B) Representative tracks of single cells cultured without (top) or with (bottom) AhRi. Scale bar represents 10 **μ**m. (C) Average transition magnitude (circle, right) and directionality (vector, left) of cells occupying the 2D morphological UMAP space. The transition magnitude was calculated as the average magnitude of all the transitions within a particular umap region, and the transition directionality was calculated as the net transition vector over all cells within that region.
(TIFF)

S11 Fig Architecture of convolutional classifier neural network for live cell classification. (TIF)

S12 Fig Architecture of convolutional variational autoencoder for cell shape and texture learning. (TIF)

S13 Fig Architecture of convolutional vanilla Auto Encoder (AE) for cell shape and texture learning. (TIF)

S14 Fig Architecture of convolutional Adversarial AutoEncoder (AAE) for cell shape and texture learning. (TIF)

S15 Fig Architectures of the generator, encoder, and discriminator module of clusterGAN for cell shape and texture learning. (TIF)

S1 Table Transition probabilities between different morphological clusters in pairwise tracking analysis. (PDF)

S1 Movie Representative timelapse brightfield movie of cultured AML cells. (MP4)
