# Supplementary figures and images for: Unsupervised discovery of dynamic cell phenotypic states from transmitted light movies
Source: PLoS Comput Biol. 2021 Dec 30;17(12):e1009626. doi: 10.1371/journal.pcbi.1009626 (PMC8754342; doi:10.1371/journal.pcbi.1009626)

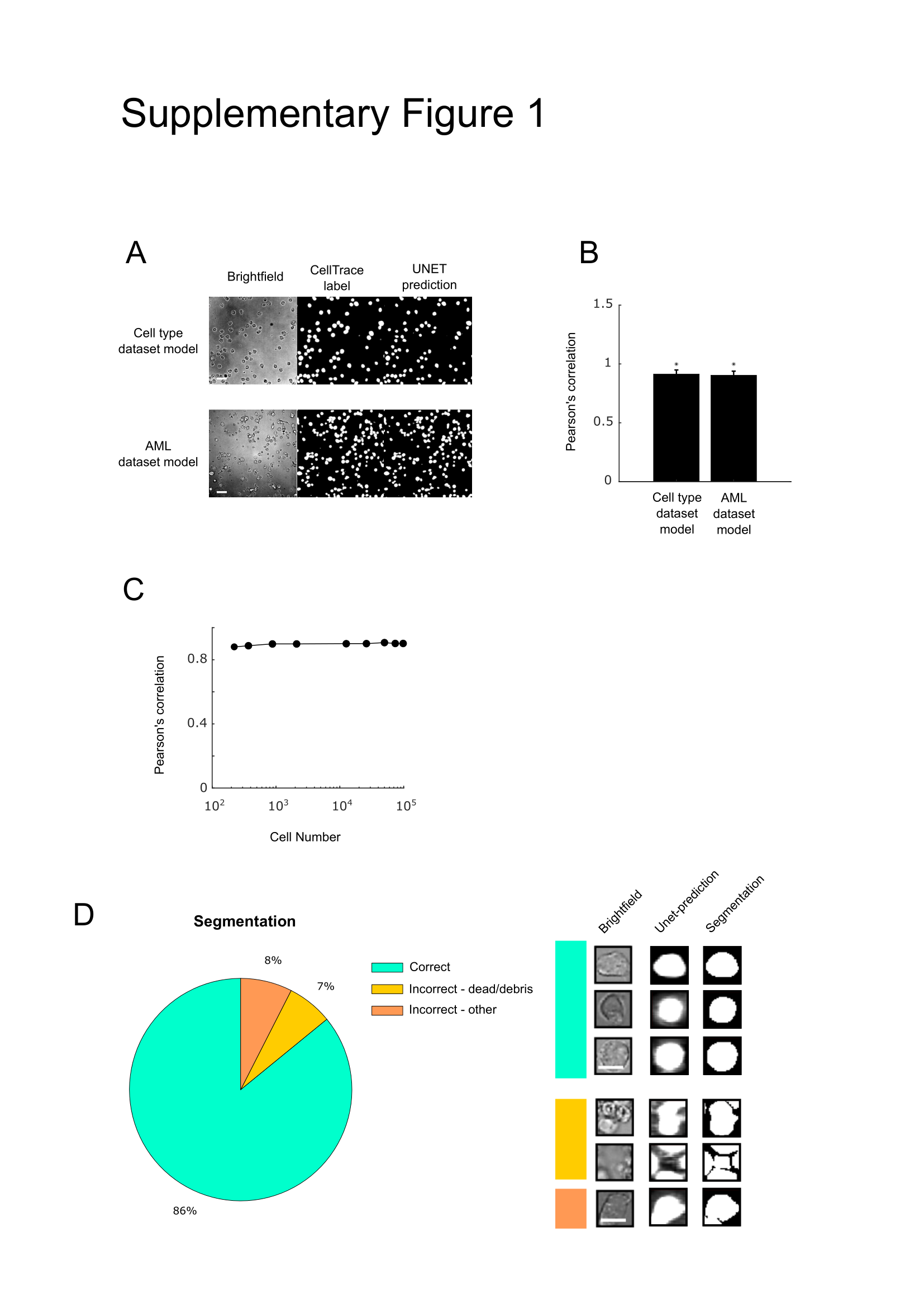

Supplement: S1 Fig — (A) Sample images from models trained on brightfield images from the cell type dataset (top) and the Acute Myeloid Leukemia dataset (bottom). Scale bar represents 20 μm. (B) Pearson’s correlation coefficients measuring correlation between the ground truth fluorescence images and predicted synthetic images. Asterisk (*) represents the theoretical upper limit of the model’s performance for each dataset. Such a model would perfectly predict the fluorescent level of each cell but not be able to predict fluorescent noise that arises from the instrumentation. (see21 for detailed method). (C) Pearson’s correlation coefficients for label-free prediction using different cell numbers for training. (D) Segmentation performance of the ictrack software (left) and sample segmented objects along with their brightfield and predicted fluorescence images (right). Scale bar represents 5 μm. (TIF) [file pcbi.1009626.s001.tif]

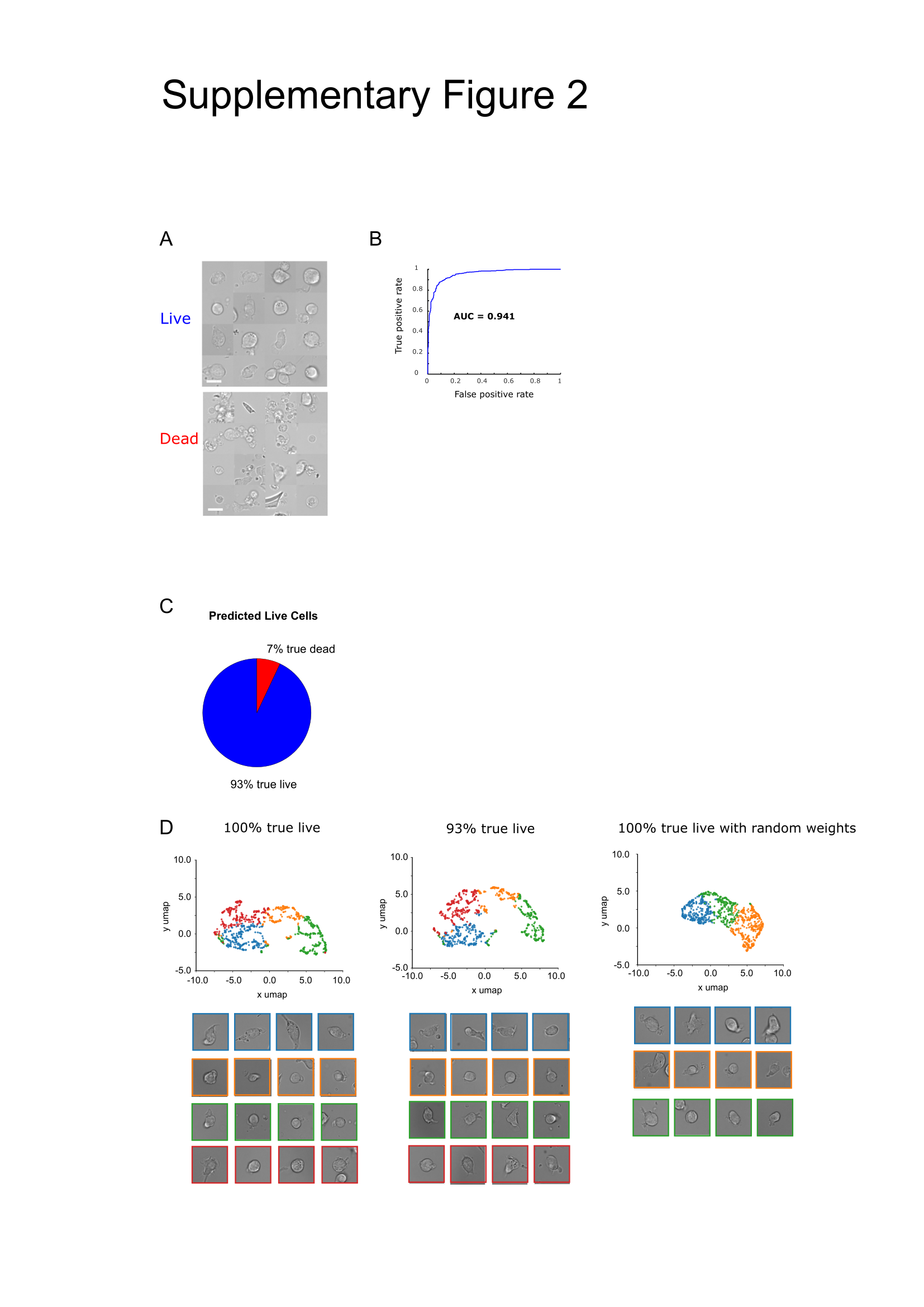

Supplement: S2 Fig — Brightfield crops of selected cells are classified as either ‘Live’ or ‘Dead’ using a convolutional classifier that is trained to recognize dead cells using a manually labeled dataset. (A) Representative cell crops classified as ‘Live’ or ‘Dead’. Scale bar represents 10 μm. (B) Receiver operating characteristic (ROC) curve measuring the prediction performance of the trained classifier. AUC: Area under the curve. (C) Fraction of correctly classified live cells out of ~1000 segmented objects. (D) UMAP plots show clustering of latent encodings of a cell population (top), with 100% confirmed live cells, 97% confirmed live cells, and 100% confirmed live cells with an unlearned variational autoencoder, where weights of neuron layers had randomized weights. Sample cell images from each identified cluster are also shown (bottom). (TIF) [file pcbi.1009626.s002.tif]

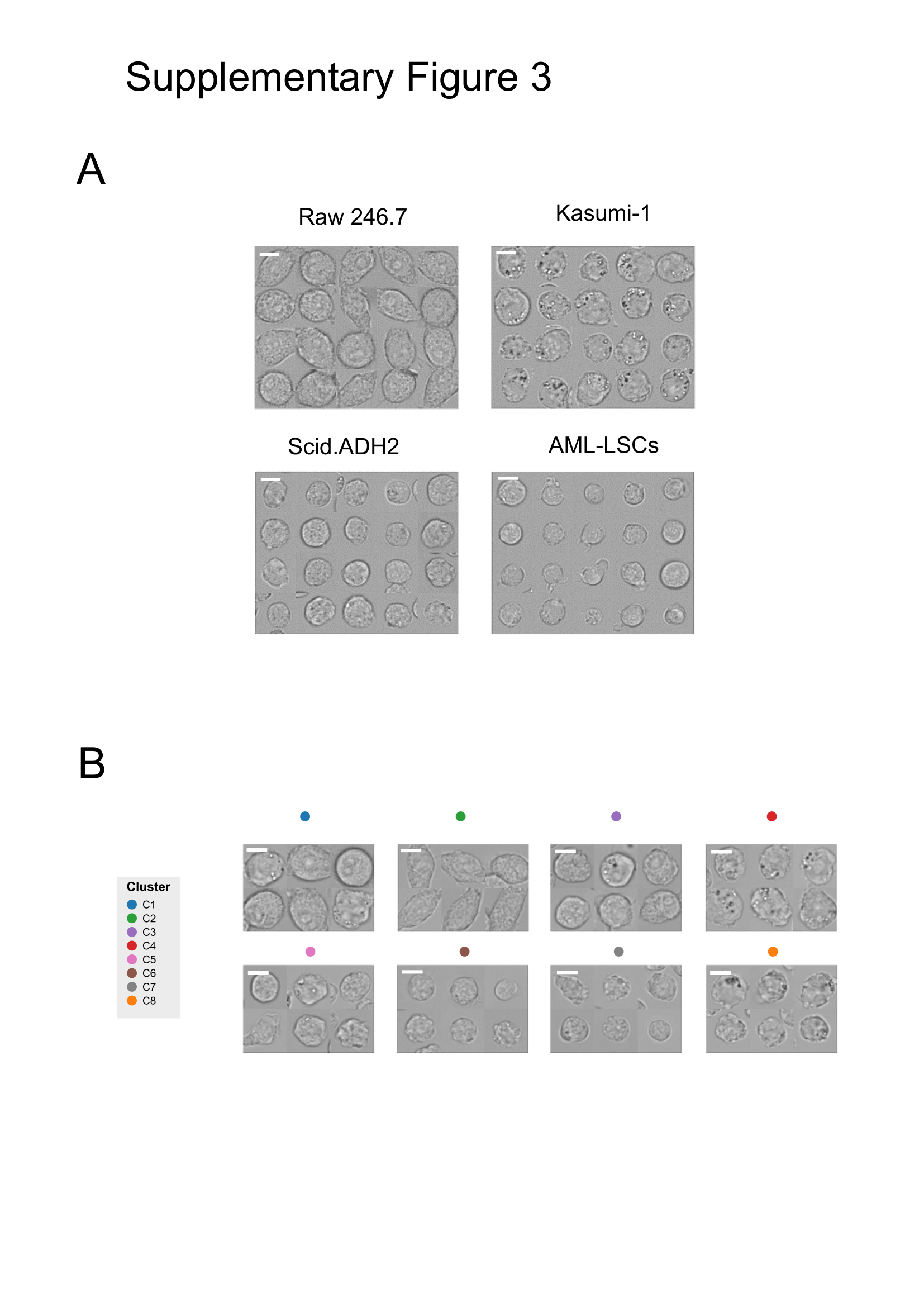

Supplement: S3 Fig — (A) Representative images from the four blood cell types Raw264.7, Kasumi-1, Scid-ADH2 and AML LSCs. (B) Representative images from eight different morphological clusters identified by Louvain clustering of the UPSIDE-generated latent vectors from each cell type. Scale bar represents 5 μm. (TIF) [file pcbi.1009626.s003.tif]

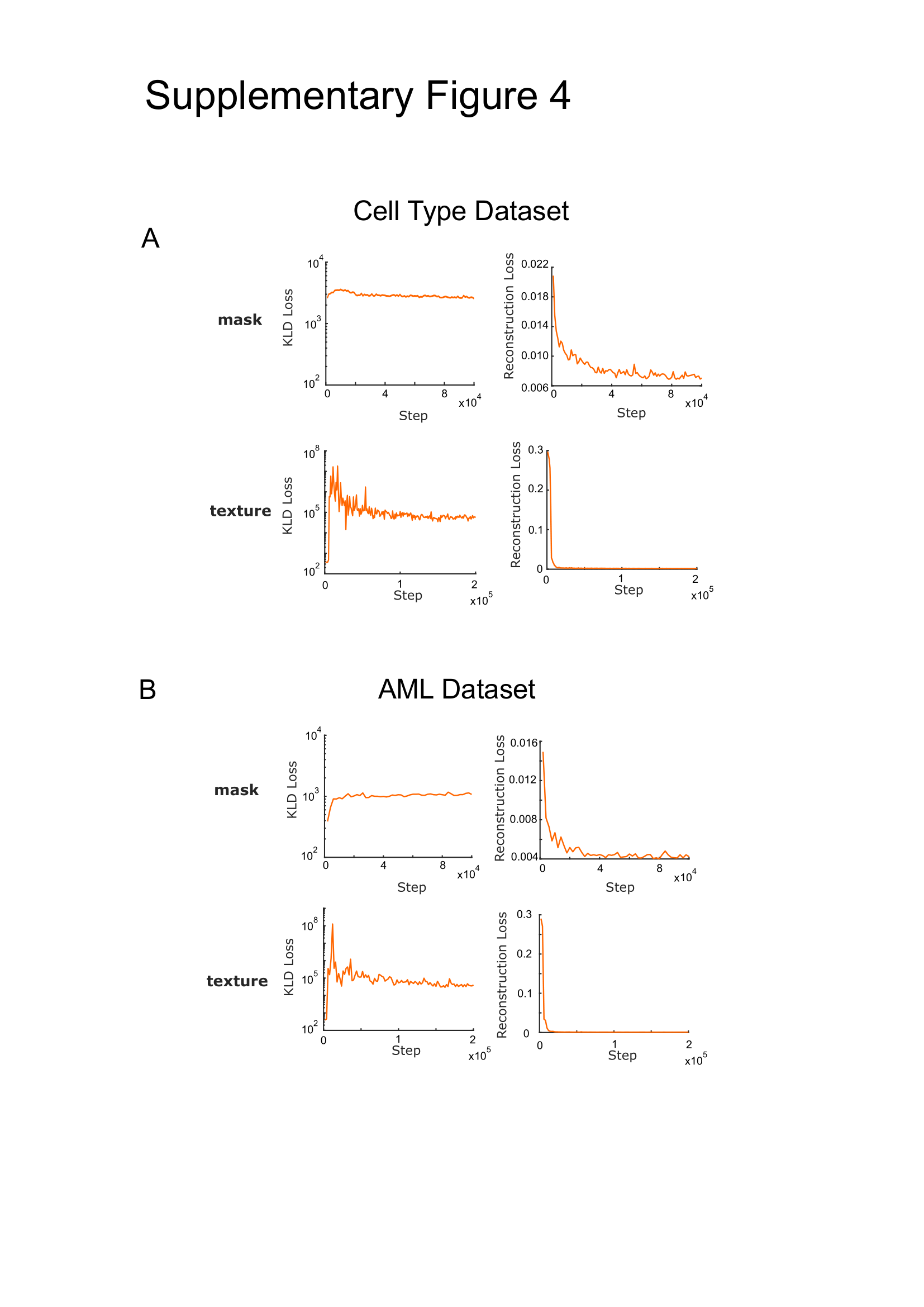

Supplement: S4 Fig — Reconstruction and Kulback-Leibler Divergence (KLD) losses of the models for the Cell Types Dataset (A) and the Acute Myeloid Leukemia Dataset (B). (TIF) [file pcbi.1009626.s004.tif]

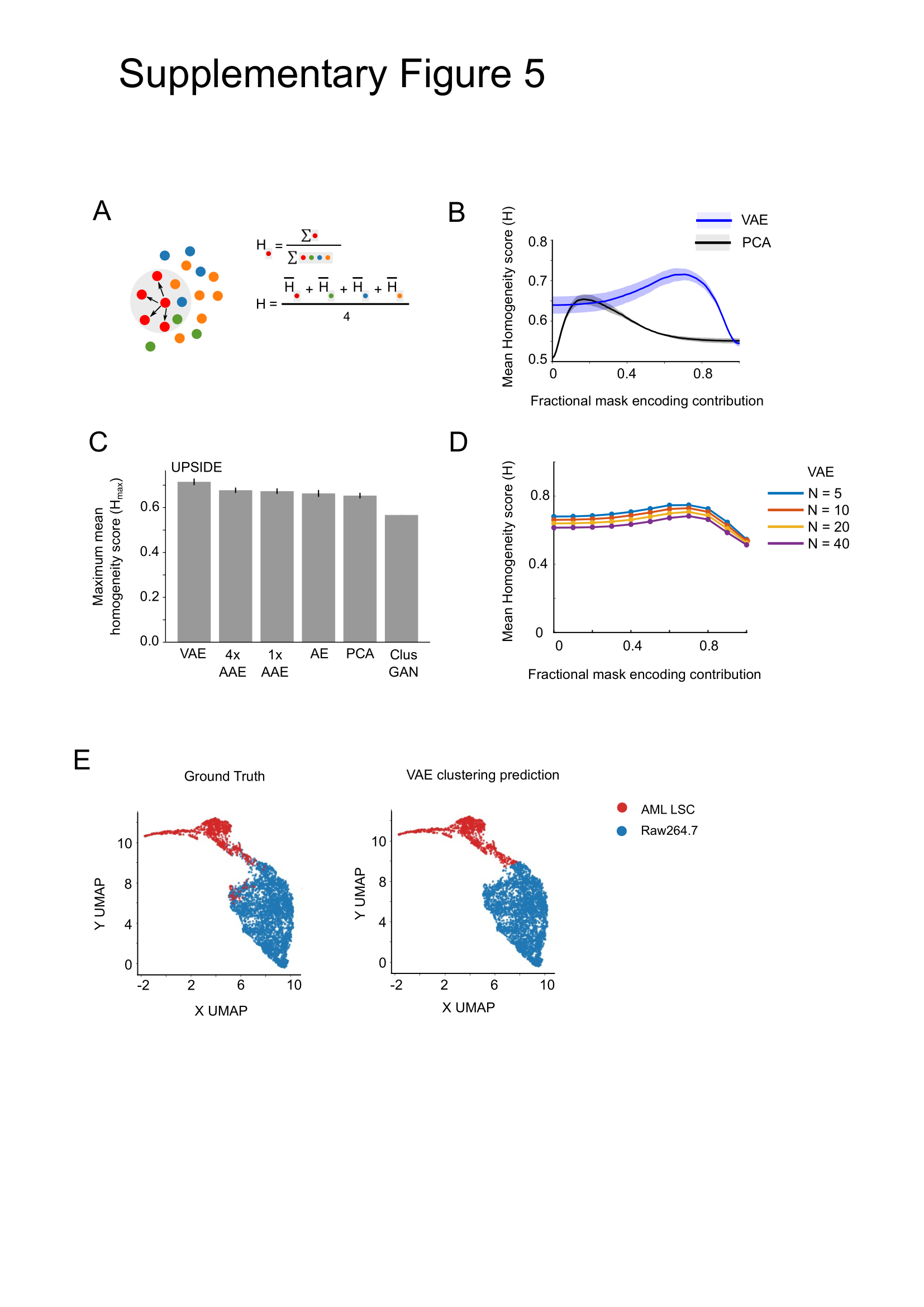

Supplement: S5 Fig — (A) The cell type homogeneity score, defined as the mean fraction of the N nearest neighboring cells of the same type as the cell of interest, averaged over all cells, measures how well different cell types are separated in latent space. (B) Mean nearest neighbor score (H) across 4 cell types obtained with different relative mask weight contribution for encodings generated by either VAE or PCA method. (C) Maximum nearest neighbor scores (Hmax) for VAE, PCA, and other alternative deep learning architectures. Hmax is defined as the highest mean nearest neighbor score across all weight combinations of mask and texture contributions. VAE: Variational Autoencoder, 4x AAE: Adversarial Autoencoder with latent dimension trained to fit a 4 mixed gaussian distribution, 1x AAE: Adversarial Autoencoder with latent dimension trained to fit a normal distribution, Clus GAN: Cluster Generative Adversarial Autoencoder with the one hot encoding component module removed, PCA: Principal Component Analysis. (D) Cell type homogeneity scores for the VAE, calculated using different numbers of neighbors N. (E). UMAP projections showing ground truth (left) and predicted clustering for the VAE (right), for two cell types, AML leukemic stem cells (LSCs) and Raw264.7 macrophages. (TIF) [file pcbi.1009626.s005.tif]

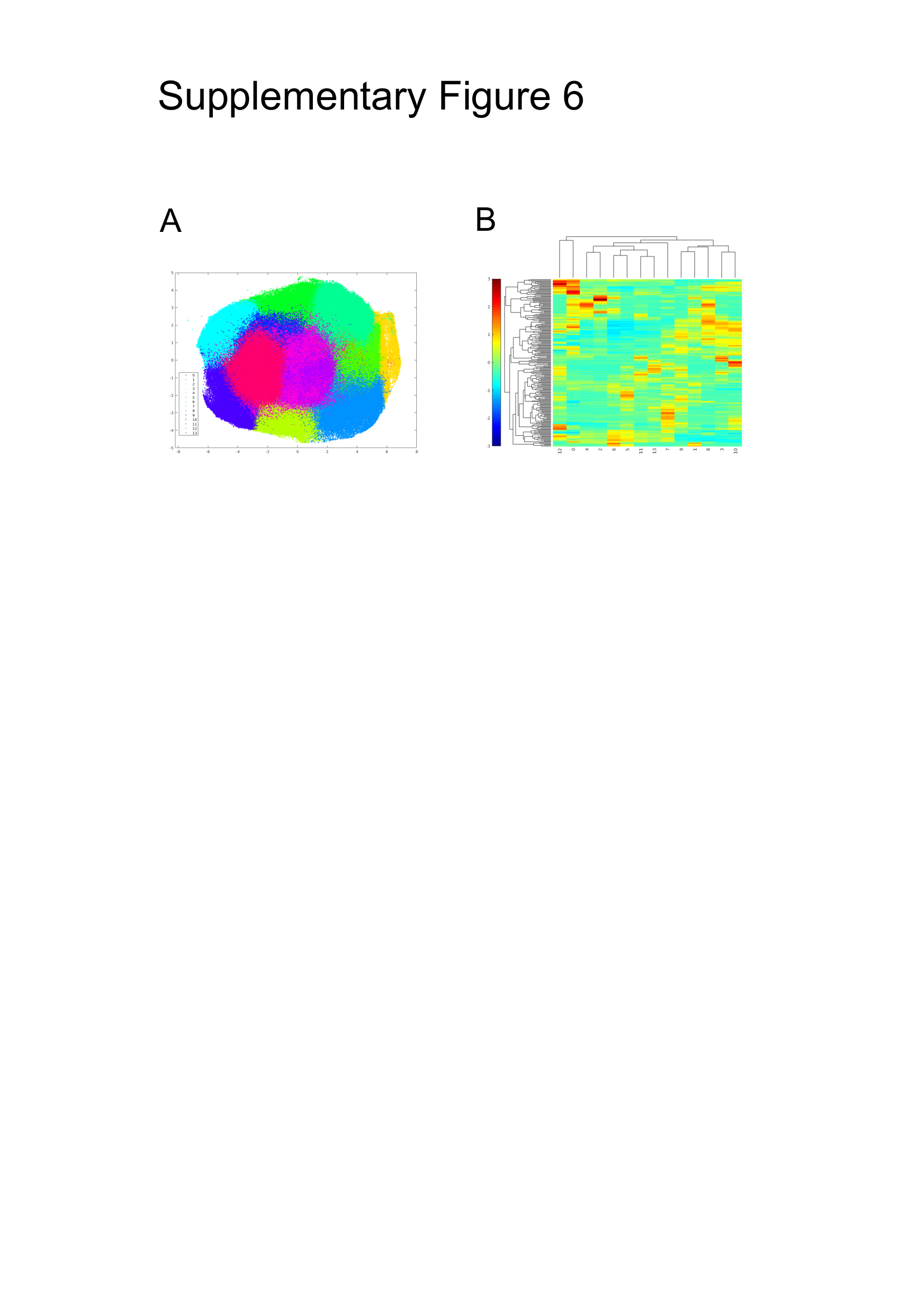

Supplement: S6 Fig — (A) 2D UMAP projection of learned mask and texture encodings from combined AML datasets. Each cell was colored based on the raw Louvain clustering result over all datasets. (B) Clustergram of the z-score from morphological groups defined by Louvain methods. Groups with closely related z-score patterns were combined into larger morphological clusters. (TIF) [file pcbi.1009626.s006.tif]

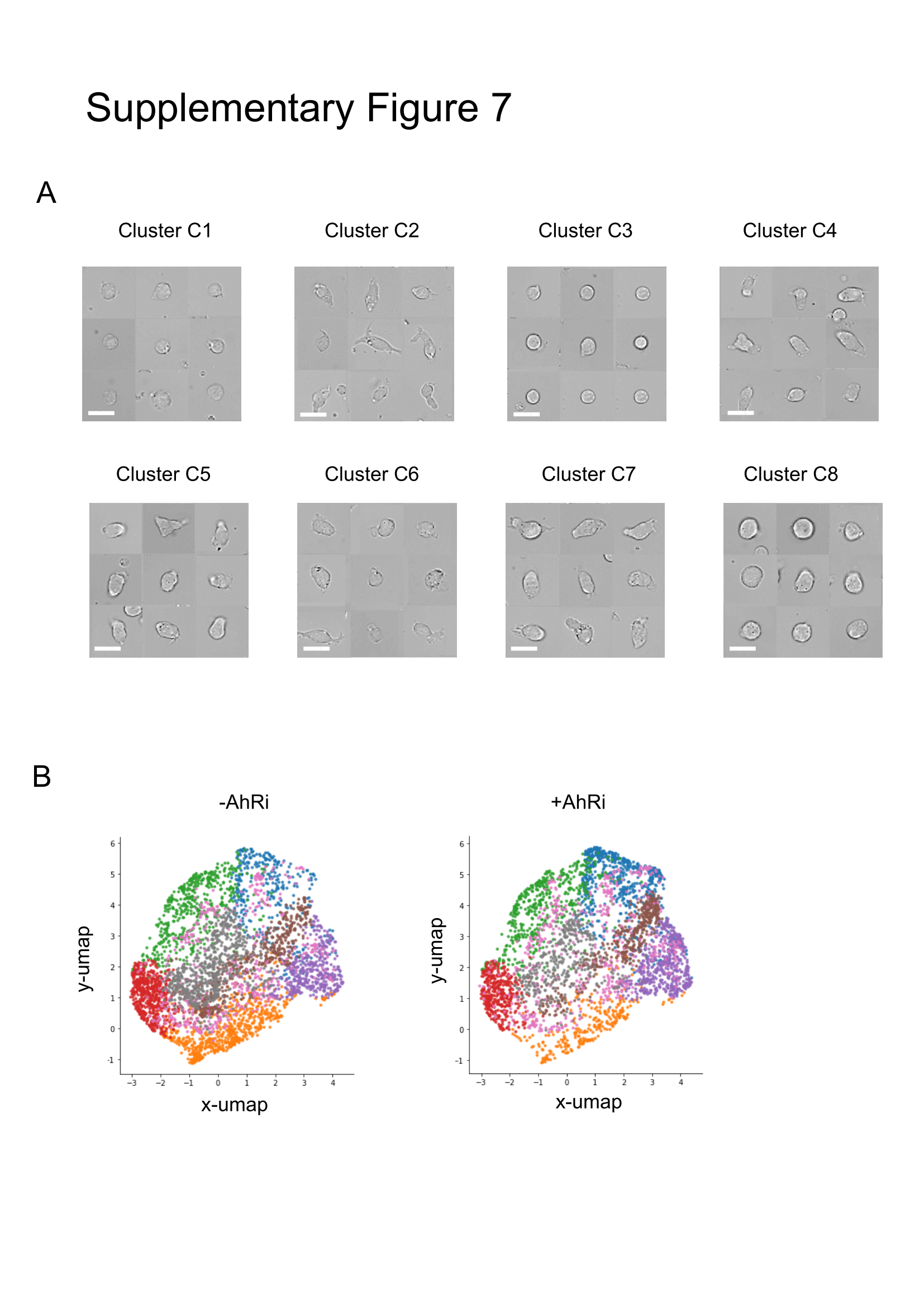

Supplement: S7 Fig — (A) Representative imags of cells in different morphological clusters. Scale bar represents 10 μm. (B) 2D UMAP projections of latent space encodings from the combined AML dataset separated into + and - AhRi conditions. (TIFF) [file pcbi.1009626.s007.tiff]

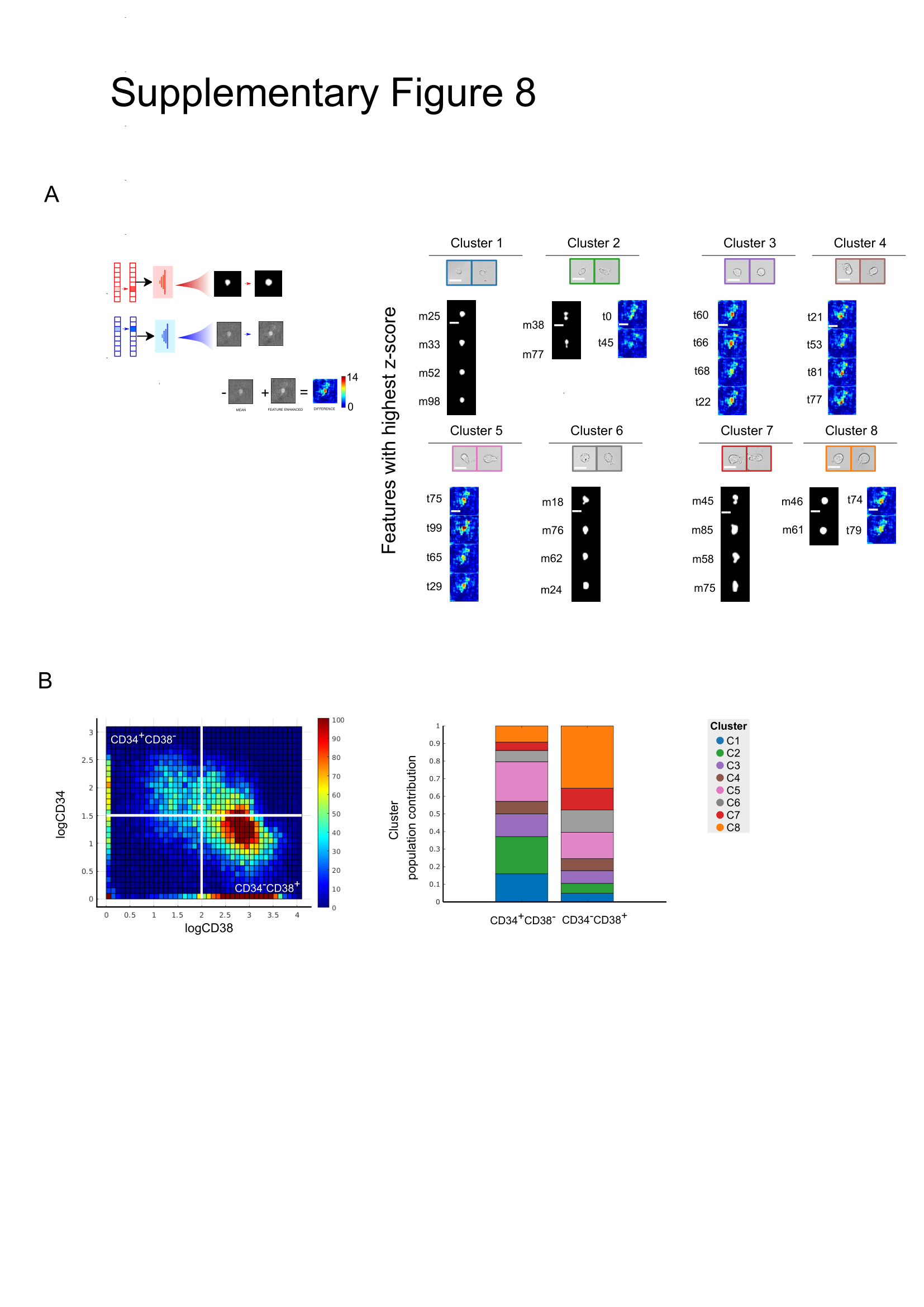

Supplement: S8 Fig — (A) Decoded texture images are accompanied by unzoomed pixel difference maps. Scale bar represents 10 μm. (B) Heatmap presenting the distribution of CD34 and CD38 expression in AML LSCs after 90hrs of culture (left). Fractional composition of each identified morphological cluster for the CD34+CD38- and CD34-CD38+ populations. (TIF) [file pcbi.1009626.s008.tif]

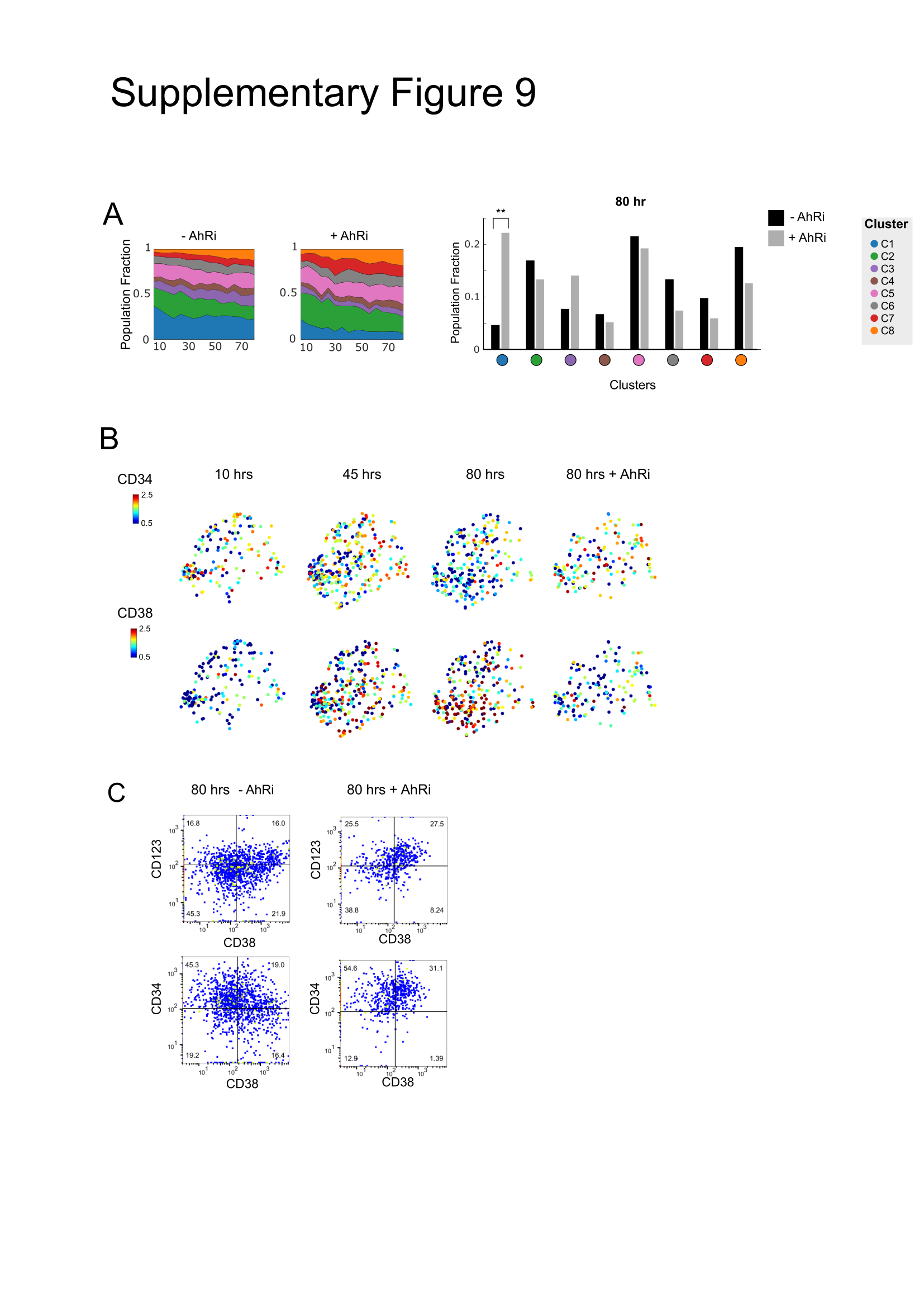

Supplement: S9 Fig — (A) Population fractions of cells in different morphological states in the absence(left) or presence of AhRi (center). Population fractions for cells in each cluster at the last time point (right). Comparisons of end-point population fractions of different morphological states, both with and without AhRi treatment, were performed using the Chi-Square test. **: p < 0.001. (B) UMAP of cells from the indicated time points. Unless otherwise indicated, cells were not treated with AhRi. Colors represent CD34 and CD38 expression levels at different time points. (C) Flow cytometry analysis of CD34, CD38, and CD123 expression levels of patient-derived AML cells cultured for 80 hrs, taken without imaging. (TIF) [file pcbi.1009626.s009.tif]

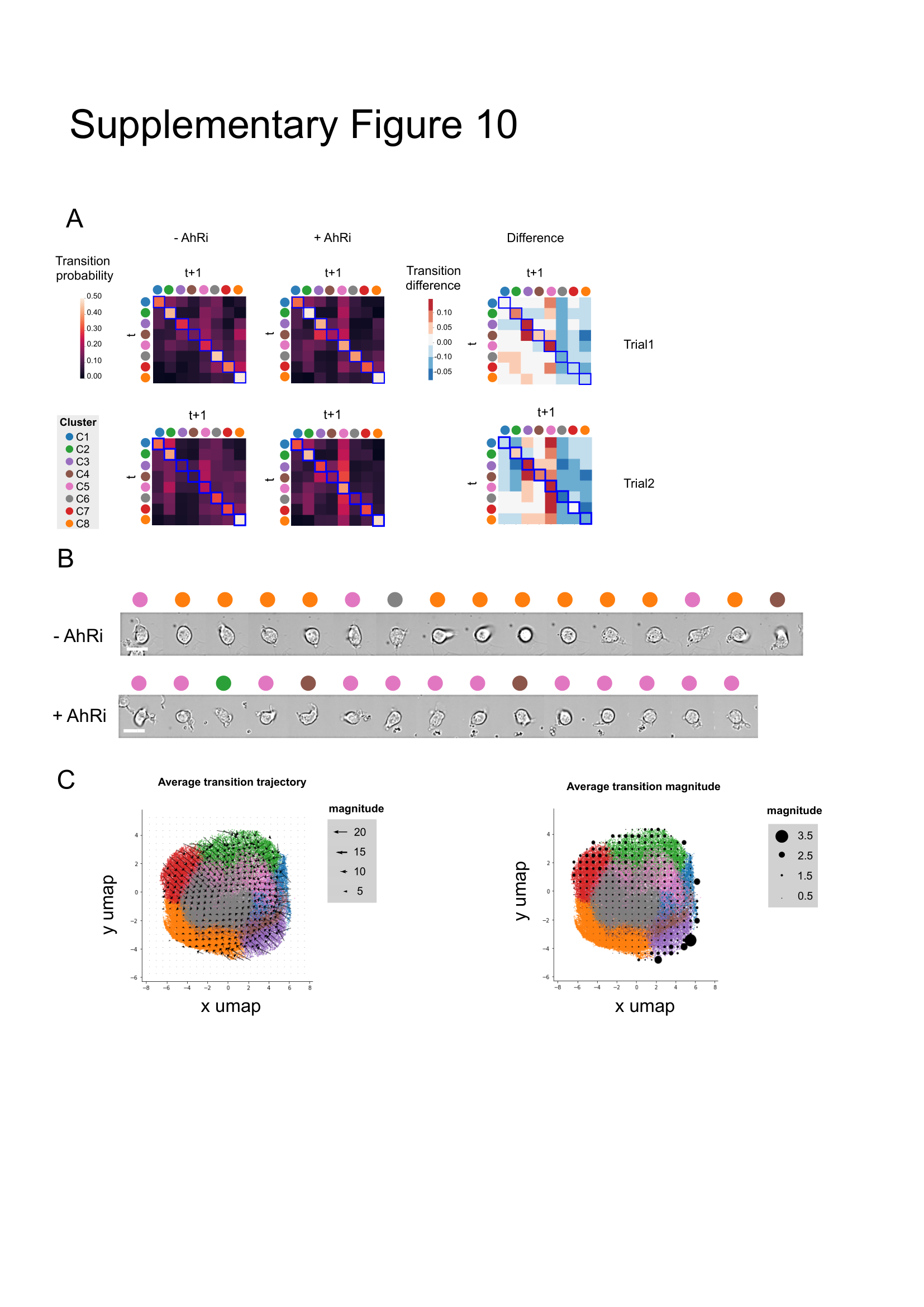

Supplement: S10 Fig — (A) Transition probability matrices between identified morphological states with and without AhRi, along with matrices showing the difference between these two conditions (right); two replicates are shown (top and bottom). (B) Representative tracks of single cells cultured without (top) or with (bottom) AhRi. Scale bar represents 10 μm. (C) Average transition magnitude (circle, right) and directionality (vector, left) of cells occupying the 2D morphological UMAP space. The transition magnitude was calculated as the average magnitude of all the transitions within a particular umap region, and the transition directionality was calculated as the net transition vector over all cells within that region. (TIFF) [file pcbi.1009626.s010.tiff]

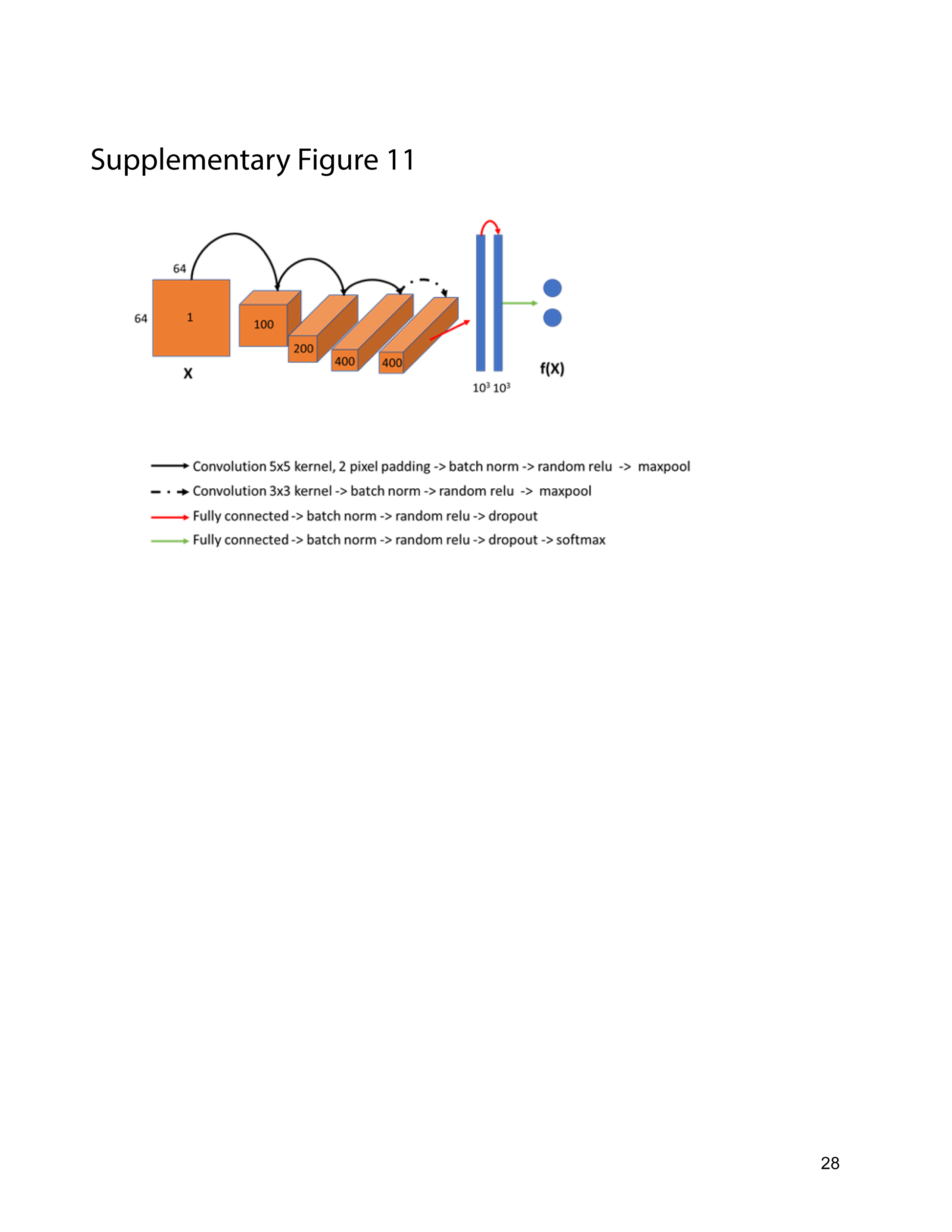

Supplement: S11 Fig — (TIF) [file pcbi.1009626.s011.tif]

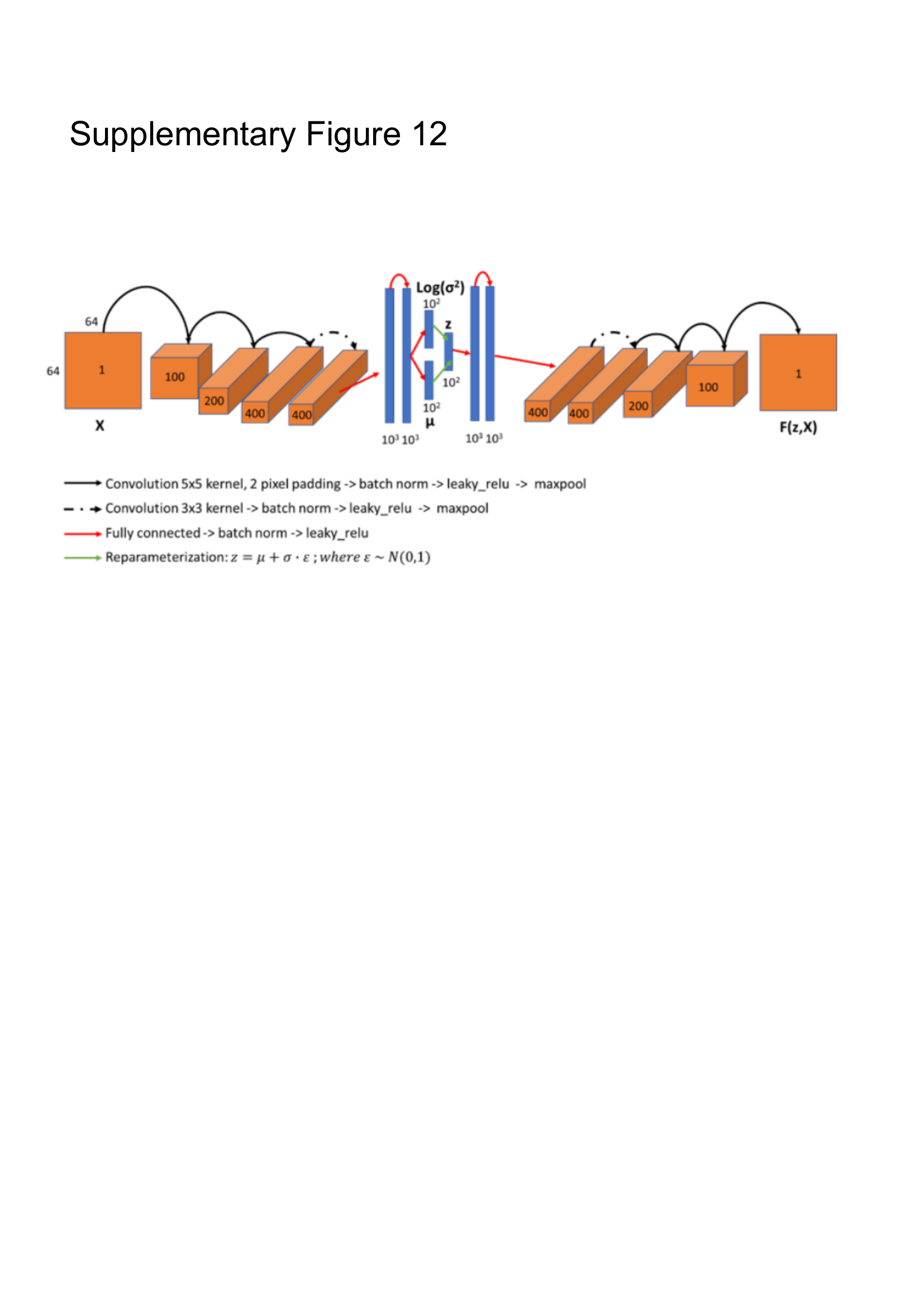

Supplement: S12 Fig — (TIF) [file pcbi.1009626.s012.tif]

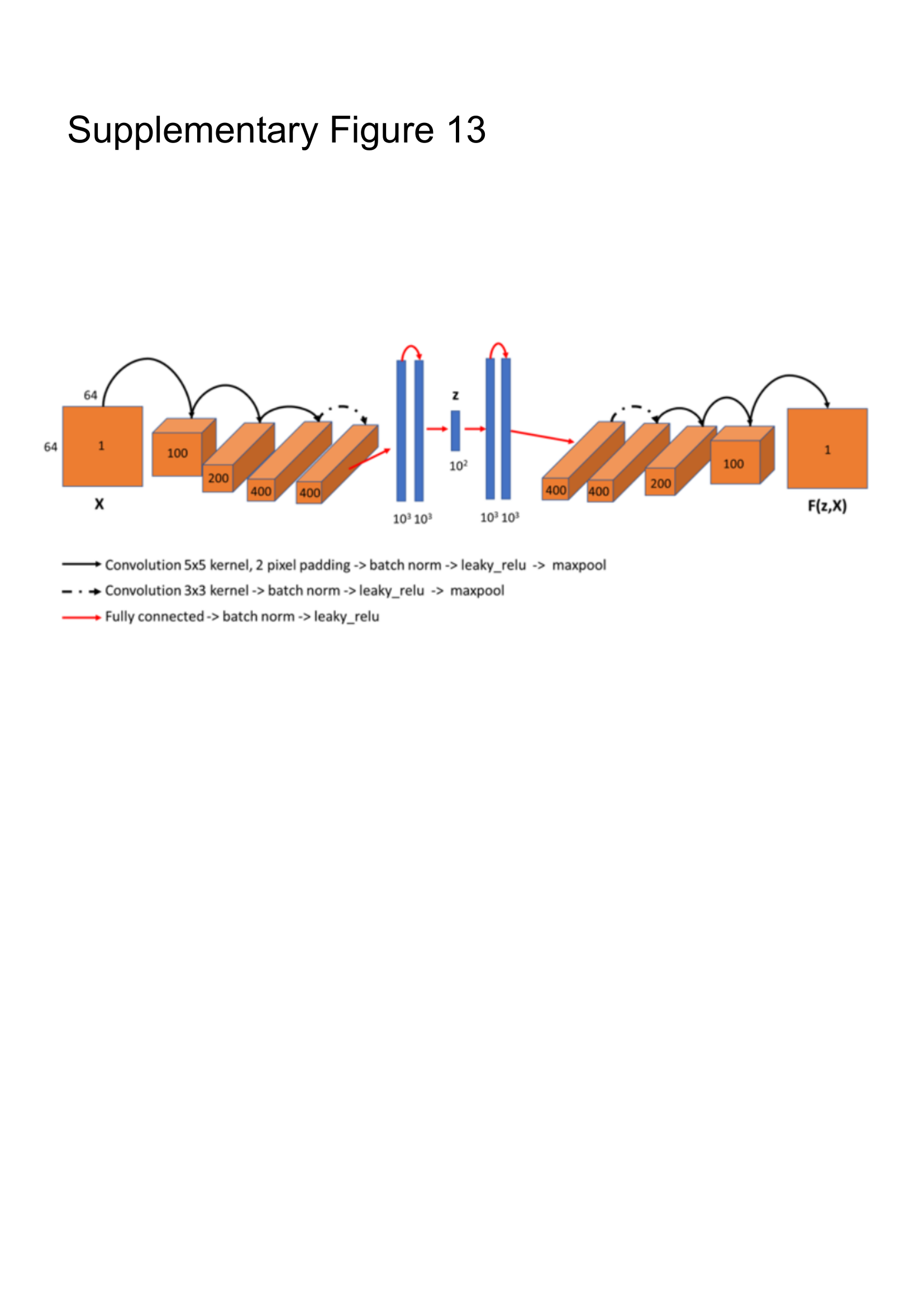

Supplement: S13 Fig — (TIF) [file pcbi.1009626.s013.tif]

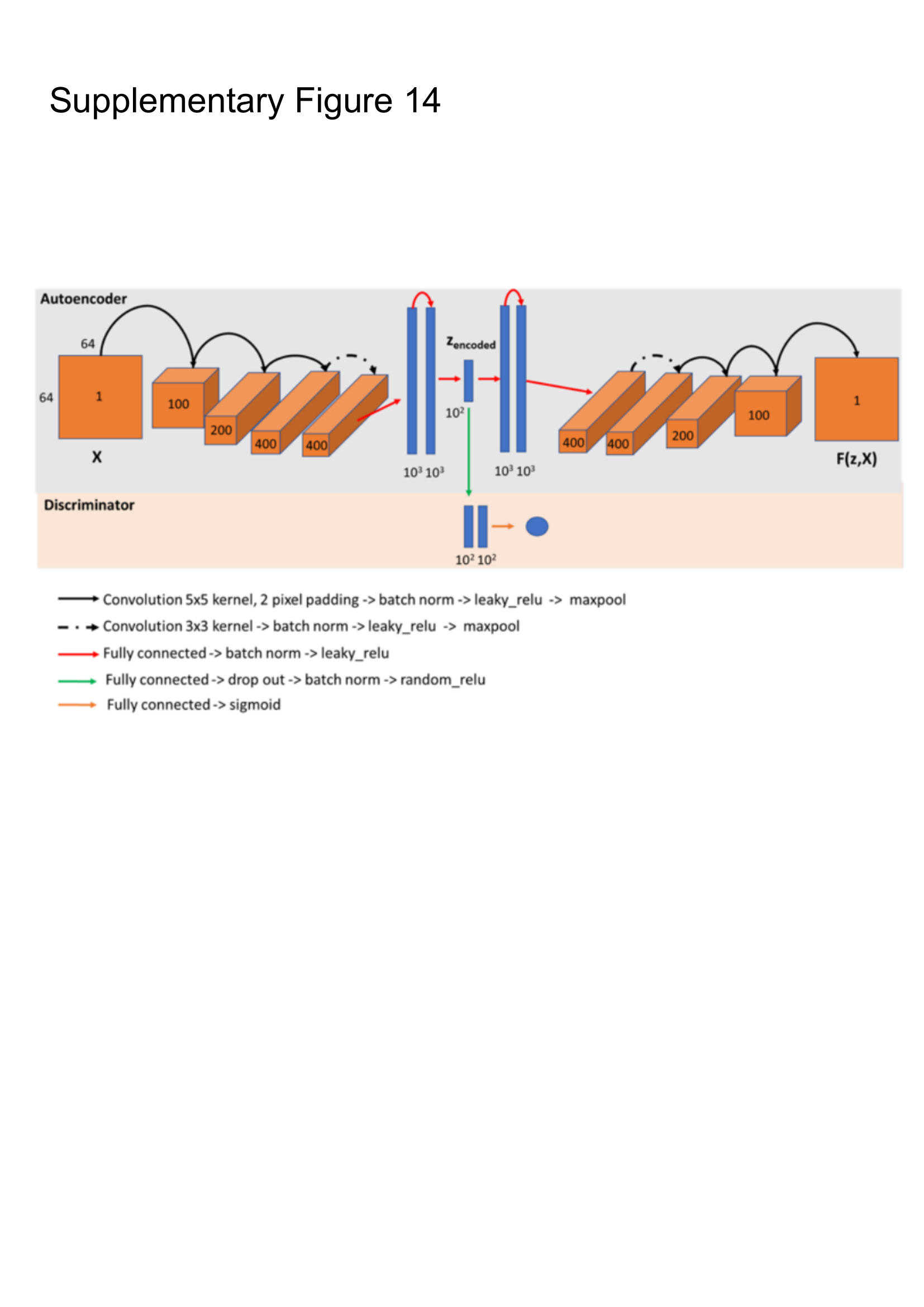

Supplement: S14 Fig — (TIF) [file pcbi.1009626.s014.tif]

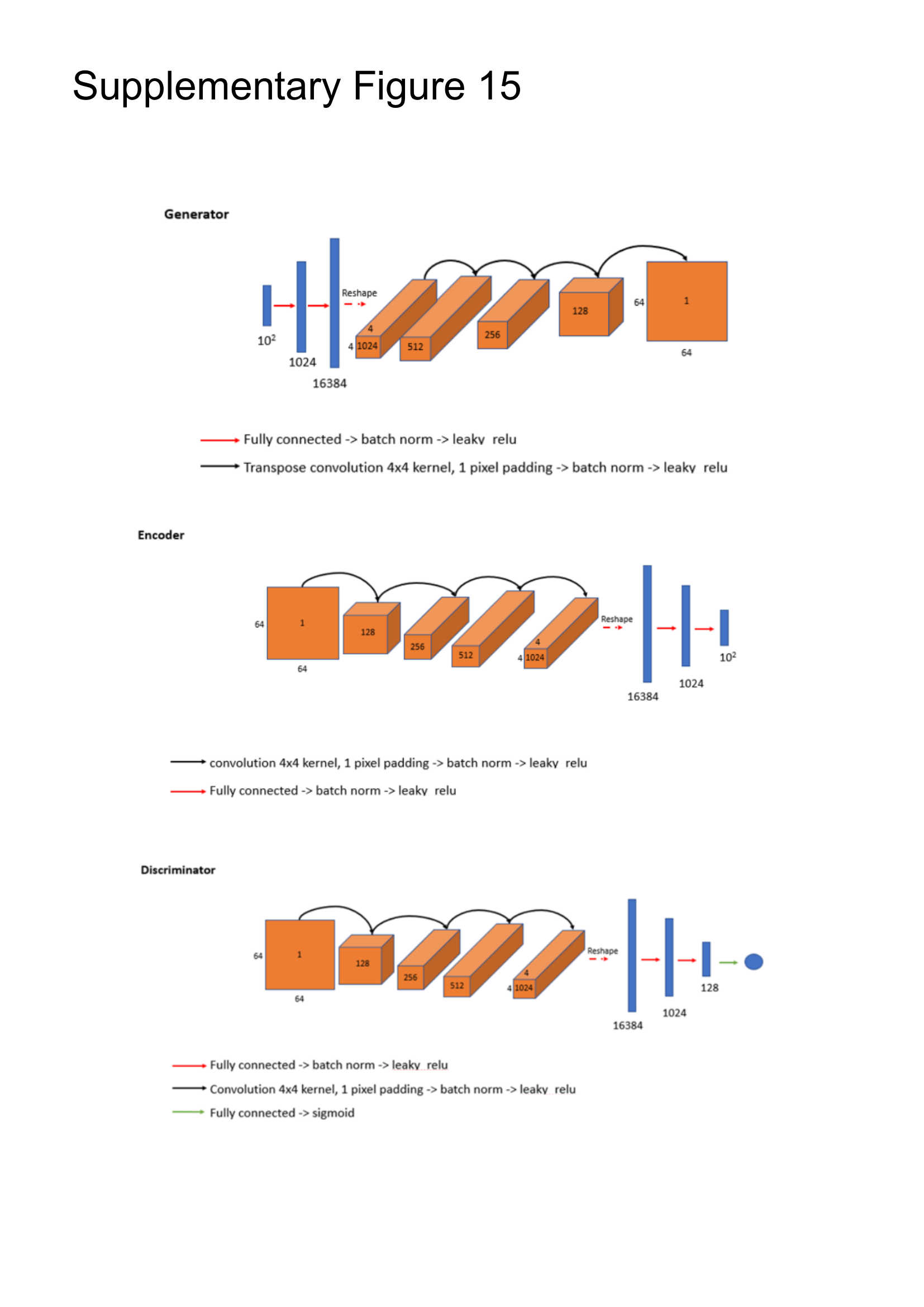

Supplement: S15 Fig — (TIF) [file pcbi.1009626.s015.tif]
