# Supplementary material for: Unsupervised discovery of dynamic cell phenotypic states from transmitted light movies
Source: PLoS Comput Biol. 2021 Dec 30;17(12):e1009626. doi: 10.1371/journal.pcbi.1009626 (PMC8754342; doi:10.1371/journal.pcbi.1009626)
Supplement: S1 Table — (PDF) [file pcbi.1009626.s016.pdf]

Table 1

Trial 1 -AhRi

	C1	C2	C3	C4	C5	C6
C1	0.34	0.17	0.12	0.05	0.11	0.15
C2	0.12	0.43	0.04	0.04	0.16	0.13
C3	0.12	0.06	0.28	0.13	0.17	0.09
C4	0.06	0.07	0.16	0.14	0.19	0.11
C5	0.07	0.13	0.11	0.08	0.27	0.13
C6	0.06	0.08	0.04	0.04	0.09	0.46
C7	0.01	0.06	0.02	0.04	0.10	0.18
C8	0.01	0.01	0.03	0.04	0.05	0.09

+AhRi

	C1	C2	C3	C4	C5	C6
C1	0.34	0.17	0.13	0.05	0.18	0.07
C2	0.11	0.51	0.03	0.03	0.21	0.06
C3	0.11	0.03	0.42	0.12	0.18	0.02
C4	0.07	0.07	0.28	0.20	0.23	0.03
C5	0.09	0.14	0.12	0.07	0.41	0.05
C6	0.10	0.13	0.03	0.03	0.11	0.40
C7	0.04	0.12	0.04	0.04	0.17	0.11
C8	0.01	0.01	0.07	0.03	0.08	0.05

Trial 2 -AhRi

	C1	C2	C3	C4	C5	C6
C1	0.34	0.25	0.05	0.04	0.10	0.14
C2	0.13	0.41	0.04	0.03	0.14	0.13
C3	0.13	0.15	0.12	0.09	0.21	0.12
C4	0.08	0.16	0.09	0.08	0.22	0.12
C5	0.08	0.22	0.07	0.07	0.23	0.11
C6	0.10	0.15	0.03	0.04	0.11	0.31
C7	0.04	0.14	0.03	0.04	0.13	0.15
C8	0.02	0.04	0.03	0.04	0.09	0.11

+AhRi

	C1	C2	C3	C4	C5	C6
C1	0.32	0.19	0.10	0.05	0.22	0.07
C2	0.10	0.44	0.03	0.03	0.24	0.07
C3	0.09	0.05	0.30	0.15	0.28	0.03
C4	0.06	0.08	0.23	0.18	0.31	0.04
C5	0.07	0.15	0.12	0.09	0.40	0.06
C6	0.12	0.17	0.08	0.04	0.27	0.16
C7	0.04	0.17	0.05	0.04	0.24	0.08
C8	0.01	0.02	0.08	0.08	0.19	0.04

C7	C8
0.02	0.05
0.06	0.02
0.02	0.13
0.06	0.21
0.08	0.14
0.10	0.15
0.36	0.23
0.08	0.70

C7	C8
0.02	0.02
0.05	0.01
0.02	0.10
0.03	0.09
0.05	0.06
0.08	0.12
0.33	0.16
0.06	0.68

C7	C8
0.05	0.03
0.09	0.03
0.09	0.08
0.12	0.14
0.13	0.10
0.14	0.12
0.31	0.17
0.15	0.52

C7	C8
0.04	0.01
0.08	0.01
0.04	0.05
0.06	0.05
0.07	0.05
0.12	0.05
0.31	0.06
0.10	0.48
